# Rare and Opportunistic Use of Torpor in Mammals—An Echo from the Past?

**DOI:** 10.1093/icb/icad067

**Published:** 2023-06-16

**Authors:** Julia Nowack, Clare Stawski, Fritz Geiser, Danielle L Levesque

**Affiliations:** School of Biological and Environmental Sciences, Liverpool John Moores University, L3 3AF Liverpool, UK; School of Science, Technology and Engineering, University of the Sunshine Coast (USC), Maroochydore DC, QLD 4558, Australia; Centre for Behavioural and Physiological Ecology, Zoology, University of New England, Armidale, NSW 2351, Australia; School of Biology and Ecology, University of Maine, Orono, ME 04469, USA

## Abstract

Torpor was traditionally seen as a winter survival mechanism employed by animals living in cold and highly seasonal habitats. Although we now know that torpor is also used by tropical and subtropical species, and in response to a variety of triggers, torpor is still largely viewed as a highly controlled, seasonal mechanism shown by Northern hemisphere species. To scrutinize this view, we report data from a macroanalysis in which we characterized the type and seasonality of torpor use from mammal species currently known to use torpor. Our findings suggest that predictable, seasonal torpor patterns reported for Northern temperate and polar species are highly derived forms of torpor expression, whereas the more opportunistic and variable forms of torpor that we see in tropical and subtropical species are likely closer to the patterns expressed by ancestral mammals. Our data emphasize that the torpor patterns observed in the tropics and subtropics should be considered the norm and not the exception.

## Introduction

Whole-body endothermy, the thermoregulatory ability of maintaining a high and stable body temperature, has been one of the key steps in the evolution of mammals and birds (Crompton et al. 1978; Bennett and Ruben 1979), as it allows animals to maintain a body temperature above ambient temperature, to be independent of ambient conditions, and to live and be active in cold habitats. Despite these advantages, the endothermic lifestyle does, however, come with much higher energetic costs than ectothermy. These can be somewhat reduced by energy savings through circadian changes in body temperatures with higher values during activity and lower values during resting (Aschoff 1963; Tattersall 2012; Levesque et al. 2023). Daily variation in body temperature can be pronounced in endotherms, especially when individuals are subject to high daily variations in ambient temperature and food availability (e.g., up to 6°C in numbats; Cooper and Withers 2004). However, by far the most effective energy-saving strategy available to mammals and birds is the lowering of metabolic rate via the use of torpor (Geiser 2004).

Torpor is generally viewed as a highly controlled mechanism of seasonal metabolic depression (Lyman et al. 1982), usually accompanied by a drop in body temperature (but see Grimpo et al. 2013 or Reher and Dausmann 2021). The literature usually differentiates between *daily heterotherms and hibernators*. Daily heterotherms are defined by expressing daily bouts of torpor with a regular but moderate reduction in energy expenditure and body temperature. Such bouts of daily torpor are often restricted to the rest phase, and animals may still maintain normal foraging activity during the active period (Ruf and Geiser 2015). Hibernation, on the other hand, is characterized by extended periods of inactivity during which energy expenditure is reduced to a fraction of that during normothermic rest and body temperature is often close to ambient temperature (Geiser 2021). During hibernation, animals undergo torpor bouts of a few days to weeks, generally interrupted by short, regular arousal periods (French 1985; Ruf et al. 2021). Typical examples of classical seasonal Northern hemisphere torpor use are shown by the Djungarian hamster (*Phodopus sungorus*), which uses bouts of daily torpor, and hibernating Arctic ground squirrel *(Urocitellus parryii)* with body temperatures below 0°C (Barnes 1989). In both species, torpor is used on a regular basis during winter, is absent during the summer, and is usually accompanied by morphological changes, such as a photoperiod-induced winter phenotype in Djungarian hamsters (Hoffmann 1973) or pronounced seasonal fattening in Arctic ground squirrels (Sheriff et al. 2013). Examples like those described above have initially led to the general idea that the ability to use torpor may have evolved as a winter-survival mechanism in response to cold habitats (Twente and Twente 1964). However, torpor is not only employed by animals living in cold and highly seasonal habitats, but also by species living in warm and/or arid climates outside of the Holarctic region (e.g., reviewed in Geiser and Körtner 2010; McKechnie and Mzilikazi 2011; Boyles et al. 2013; Nowack et al. 2020). Interestingly, torpor patterns in non-Holarctic species are often more variable than those commonly observed in Northern temperate zones, and torpor expression can range from shallow torpor (Shankar et al. 2022; Nowack et al. 2023) or very short micro-torpor bouts (Reher et al. 2018) to extensive torpor bouts of a few months without regular arousal phases (Dausmann et al. 2004; Dausmann et al. 2020; Lovegrove et al. 2014). Furthermore, torpor expression is often more flexible than previously assumed, and individuals of one species or population may differ in their use of *torpor* and either employ extended periods of inactivity with a series of multiday or weeklong torpor bouts (*hibernation*), multiday bouts of torpor lasting a few days (*prolonged torpor*), or short bouts of torpor, similar in duration to the bouts of daily torpor described for daily heterotherms (reviewed in Nowack et al. 2020; also see Glossary for definitions). Australian Eastern pygmy possums (*Cercartetus nanus*), for example, commonly undergo hibernation during winter, but individuals vary in their torpor pattern, and some individuals may only use short or prolonged bouts of torpor and do not undergo hibernation (Turner et al. 2012). Furthermore, male possums may also use opportunistic short bouts of torpor during the summer, possibly in response to a negative energy balance due to prioritizing searching for females over food acquisition (Turner et al. 2012). Importantly, such opportunistic use of torpor enables non-Holarctic animals to respond to acute emergencies and has been described to be triggered in response to fast-scale natural disasters, such as droughts, fires, storms, or floods (reviewed in Nowack et al. 2017) and less predictable climates in subtropical and tropical areas also coincide with more variable durations of torpor bouts (reviewed in Geiser 2020; Nowack et al. 2020).

The widespread occurrence of torpor in all three mammalian subclasses (monotremes, marsupials, and placentals) suggests that it is an evolutionarily old trait and that endothermy evolved via a heterothermic ancestor that likely showed high variability in body temperatures including some form of torpor use (Grigg et al. 2004; Lovegrove 2012; Ruf and Geiser 2015)—a view that is now widely accepted among scientists. As early mammals evolved under conditions more similar to species in the tropics, this has led to the idea that flexible use of torpor may be the ancestral form that would have been used by early mammals at the transition to the whole-body endothermy found in extant mammals and birds (Grigg et al. 2004; Lovegrove 2012). As global temperatures cooled during the Cenozoic, more constrained, predictable forms of torpor would have been made possible (Lovegrove 2012; Lovegrove 2017). If this is the case, one would therefore expect opportunistic torpor use to be widespread and not restricted to species in non-Holarctic regions, and seasonal, programed torpor to be restricted to temperate and polar climates.

To evaluate biogeographical and phylogenetic patterns of torpor use in mammals, we collated datasets on mammals known to use torpor. We noted the types of torpor used, the season(s) in which torpor has been recorded, as well as if the animals needed significant, i.e. weeks or months’, of preparation time before entering hibernation. We hypothesized that non-seasonal and flexible use of torpor is the ancestral condition in mammals and would therefore be found across multiple taxa and worldwide, and further predicted that strictly seasonal torpor expression that requires preparation would be restricted to cold and/or highly seasonal environments and only a few taxa.

## Methods

We based our dataset on comprehensive review articles (Ruf and Geiser 2015; Levesque et al. 2016; Nowack et al. 2020) and ran a systematic literature search to update these with the records of new (or previously missed) discoveries to compile a list of mammal species known to use torpor to date (see Supplementary Table S1). To obtain a standardizable proxy for a species’ geographical range, we added mid-range distribution (latitude and longitude) for each species based on entries in the mammal life history database PanTHERIA (Jones et al. 2009). Data on the distribution for 10 species missing from PanTHERIA were obtained from the International Union for Conservation of Nature (IUCN) Red List and were downloaded as shapefiles (IUCN 2022), from which the centroid was calculated using the “st_centroid” function in the R package “sf” (Pebesma 2018).

**Table:** Glossary

Term	Definition used in this paper
*Daily heterotherm*	Animals that only display bouts of *daily torpor* lasting less than 24 hours (in contrast to *hibernators*).
*Daily torpor*	Short bouts of torpor of less than 24 hours; employed by *daily heterotherms*.
*Flexible torpor use*	Flexibility in torpor pattern with individuals being able to switch between torpor types or individuals of a population adapting different torpor types, even in response to the same environmental conditions.
*Heterothermy*	Fluctuations in body temperature and metabolic rate seen in endothermic animals.
*Hibernator*	Animals that undergo *hibernation*.
*Hibernation*	Sequence of multiday torpor bouts interrupted by periodic arousal and short normothermic periods of several hours; restricted to the hibernation season (several weeks to months) and without extended activity and foraging.
*Opportunistic torpor*	Spontaneous and flexible torpor use that can occur at any time of the year in a flexible manner.
*Prolonged torpor*	Multiday torpor bout after which animals are normothermic and active for days before potentially undergoing additional bouts of short or prolonged torpor; Occurs independent of hibernation.
*Seasonal torpor*	Torpor use is restricted to a single season, usually the cold or the dry season.
*Short torpor*	Short bouts of less than 24 hours employed by animals that can also undergo longer multiday torpor bouts (*prolonged torpor* or *hibernation*); bouts are occurring at any time of the year and independent of the hibernation season

We used the mid-range latitudes to classify each species as belonging to one of four *climate zones* (*tropical*: 0–23.5°N/S, *subtropical*: 23.5–40°N/S, *temperate*: 40–60°N/S, or *polar*: 60–90°N/S). We lacked mid-range coordinates for the following two species: *Mus musculus* (a commensal species with a worldwide distribution) and one unnamed species *Gerbillus species 1*. However, to include these species in the analysis, we classified them as subtropical*: Mus musculus* based on their evolutionary origin (Boursot et al. 1996) and *Gerbillus species 1* based on the locations of the study describing torpor use for the species (Gyhrs et al. 2022).

We then synthesized the following information for each species in our dataset via an extensive literature search (Supplementary Table S1): maximum torpor bout duration, seasonality, and torpor predictability. Torpor bout duration was classified as either *daily torpor* (longest duration <24 h), *prolonged torpor* (multiday bouts interspersed by extended activity), *hibernation* (sequence of multiday bouts), or data deficient, if no conclusive information about torpor bout duration was available. Seasonality of torpor use was classified as *seasonal* if torpor use was restricted to one season such as the cold or dry period, *non-seasonal* if it was seen in different seasons, or *data deficient* if this information was missing or a species was only investigated in one season or under constant laboratory conditions. Torpor predictability was defined as *predictable* when all individuals used torpor in the same way, *unpredictable* when they differ in their likelihood to enter torpor or the pattern of torpor they are using, or *data deficient* if this information was missing. We also noted whether a hibernating species was known to prepare for hibernation (food hoarding or fattening; yes/no) and whether a species that hibernates may be flexible in their use of torpor and use short torpor bouts independent of hibernation. Missing information was again listed as *data deficient*.

To estimate the ancestral character state for various parameters (seasonality, predictability, and torpor bout duration), we used the “ace” function in the R package “ape” (Paradis and Schliep 2019) with a phylogeny trimmed from the Upham et al. (2019) mammal supertree. For discrete characteristics, such as the ones used in this study, the function provides a maximum-likelihood estimation of the state at each node following Pagel (1994).

## Results

### Global distribution and type of torpor use

**Fig. 1:**
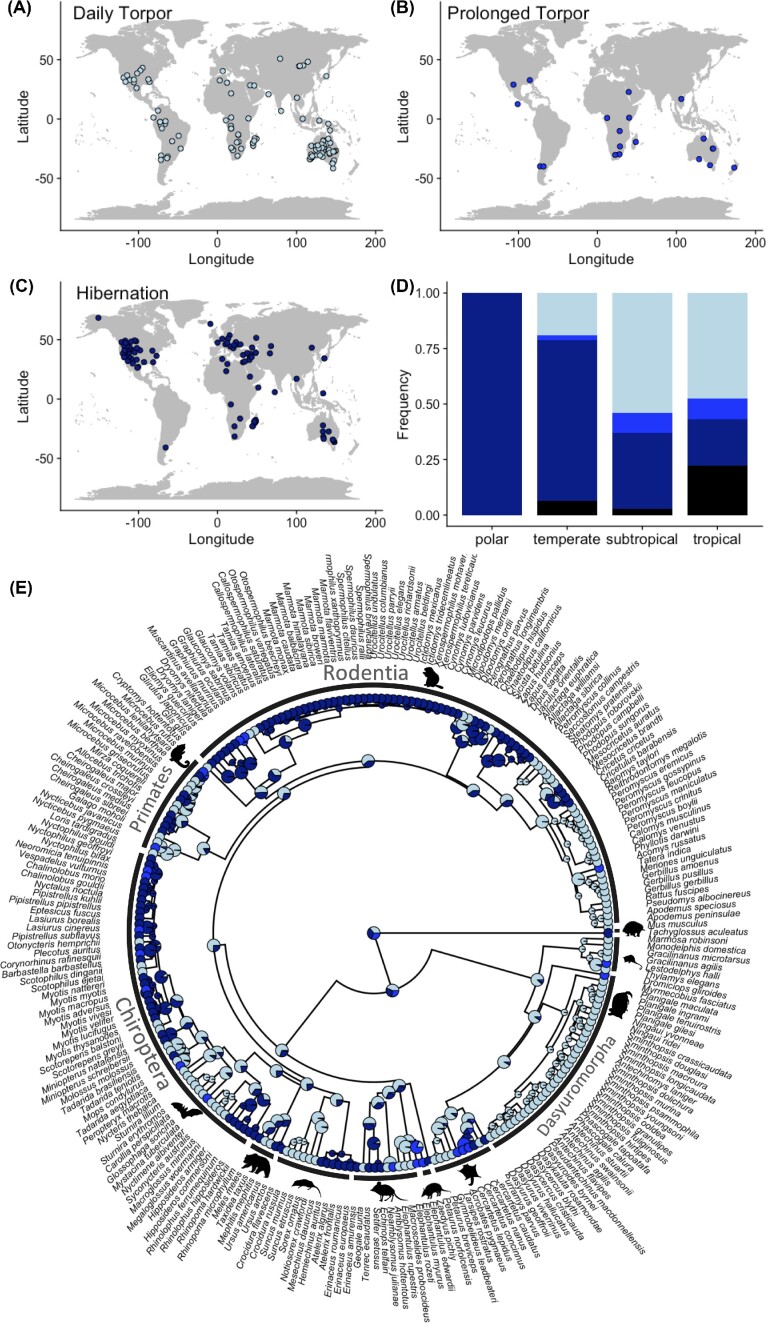
Global distribution of torpor patterns, based on the maximum duration of bouts. (A) Daily torpor (*n* = 117); (B) Prolonged torpor (*n* = 21); and (C) Hibernation (*n* = 109). (D) Frequency distribution of torpor patterns (maximum duration) in relation to the four climate zones (tropical: 0–23.5°N/S, subtropical: 23.5–40°N/S, temperate: 40–60°N/S, or polar: 60–90°N/S). Black: data deficient (*n* = 28); dark blue: hibernation; middle blue: prolonged torpor; and light blue: daily torpor. Sample sizes for the four climate zones are: polar (*n* = 2), temperate (*n* = 60), subtropical (*n* = 118), and tropical (*n* = 95). (E) The phylogenetic relationships of species with known torpor use type. The tip labels represent the longest torpor type observed in the species, using the same color scheme as in (D). Pie charts at the nodes were estimated using ancestral state estimation, and the proportion of each color represents the likelihood that the shared ancestor used that particular torpor type.

The full dataset spans 275 species and includes one species of monotreme, 57 species of marsupials, and 217 placental species (Supplementary Table S1). Species that use torpor were found in all four climatic zones, with the vast majority in subtropical and tropical areas (*n* = 213 vs. *n* = 62 for polar and temperate). Daily heterotherms, employing torpor bouts of <24 h as the longest bouts, made up 42% (117/275) of the species in our dataset. Prolonged torpor was the maximum duration of torpor bouts for 8% (21/275) of the species, and 40% (109/275) were found to undergo hibernation. For 10% (28/275) of the species, the actual pattern of torpor use remains unclear (see Supplementary Table S1). Hibernation was found in many species in polar and temperate habitats but was much less common in subtropical and tropical species (Fig. 1). Daily torpor, on the other hand, was more common in warmer climate zones and absent in the polar regions (Fig. 1).

All torpor types, including prolonged torpor, were found across the mammal phylogeny. Use of a single torpor type only was found in three clades: The dasyurid marsupials (Order: Dasyuromorpha) and shrews (Family Soricidae) have so far been found to only use daily torpor, whereas the sciurid subfamily Marmotini (chipmunks and ground squirrels) all use hibernation. The presence of prolonged torpor and hibernation in other marsupial sister clades (including other Australian marsupials) increased the likelihood of other torpor types at the ancestral marsupial node (Fig. 1E). Ancestral character estimation indicated an almost equal likelihood of any of the three torpor types being the ancestral condition (scaled likelihoods at the root of 0.35 for daily torpor, 0.33 for hibernation, and 0.32 for prolonged torpor, Fig. 1E).

### Seasonality of torpor use

We could not find information on the seasonality of torpor use for 59% (161/275) of the species. In the 41% (114/275) of species for which information about the seasonality of torpor use was available, strict seasonality was relatively rare (39%; 44/114). Torpor use was found to be used in several seasons, i.e. *non-seasonal* in 61% (70/114) of species that were investigated in several seasons.

**Fig. 2:**
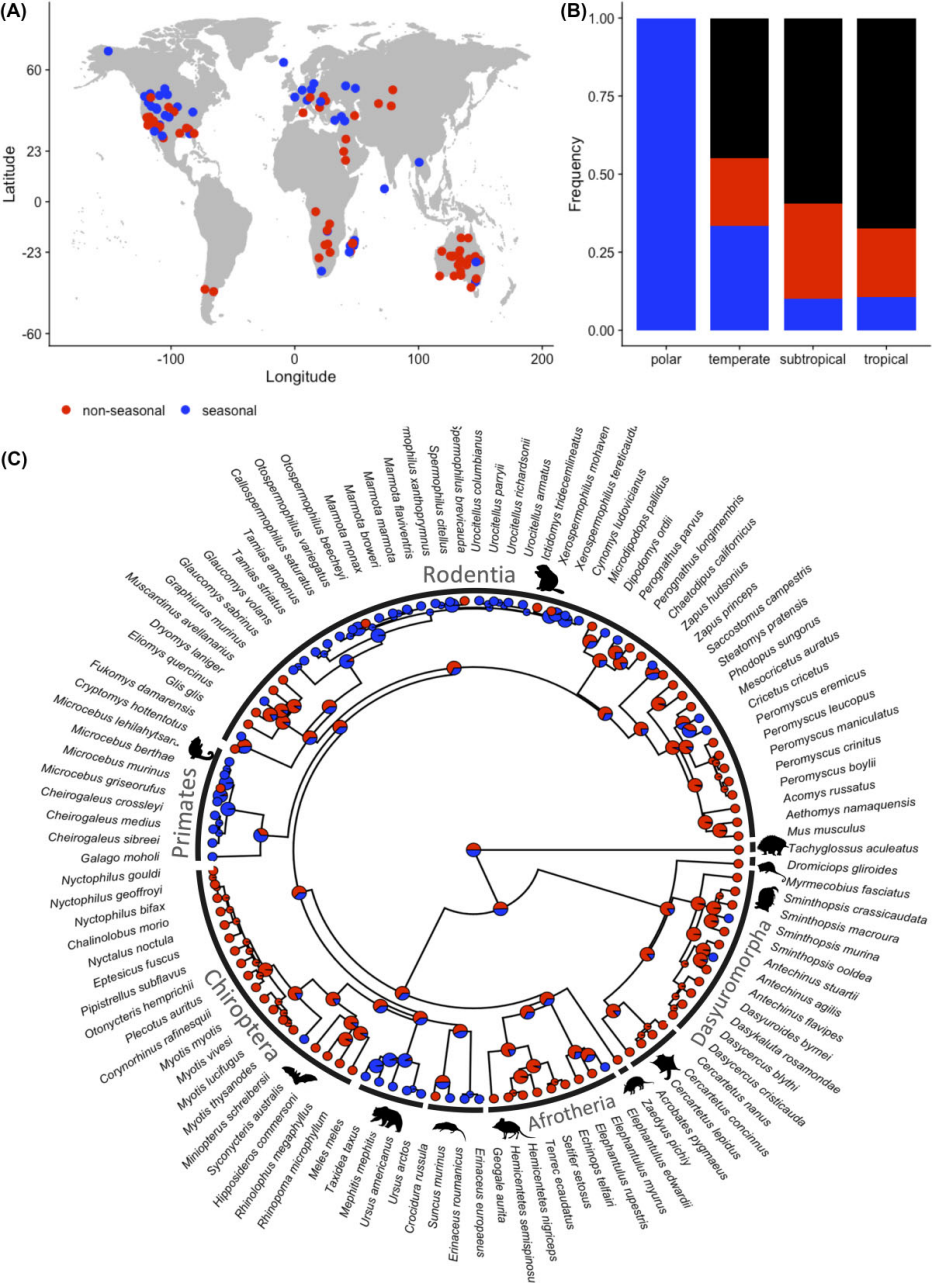
(A) Global distribution of seasonality in torpor use and whether torpor is used seasonal (blue, *n* = 44/275) or non-seasonal (red, *n* = 70/275); data deficient is not depicted (*n* = 161/275). (B) Frequency distribution of seasonality of torpor use based on climate zone. Blue: strictly seasonal use; red: torpor use not restricted to one season; black: data deficient. Sample sizes for the four climate zones (tropical: 0–23.5°N/S, subtropical: 23.5–40°N/S, temperate: 40–60°N/S, or polar: 60–90°N/S) are polar (2), temperate (60), subtropical (118), and tropical (95). (C) The phylogenetic relationships of species with data on torpor use over multiple seasons. Pie charts at the nodes were estimated using ancestral state estimation, and the proportion of each color represents the likelihood that the shared ancestor used non-seasonal (red) or seasonal torpor (blue).

Only ∼10% (22/213) of subtropical and tropical species are known for their strictly seasonal torpor use, in contrast to 36% (22/62) in polar and temperate zones regions (Fig. 2). Non-seasonal use of torpor was found in 22% of species using daily torpor (26/117), 28% of species using hibernation (30/109), and in 48% of species showing prolonged torpor (10/21) and was not restricted to subtropical and tropical habitats (Fig. 2). Only 6% (7/109) of hibernators—all species from subtropical and tropical regions—do not show seasonal fattening or food hoarding (Supplementary Table S1).

About 38% (41/109) of species that are known to undergo hibernation were found to also use opportunistic *short bouts of torpor* or *prolonged torpor* without prior preparation in response to acute negative energy balance throughout the year. This use of short bouts of torpor by hibernators was found in higher frequency (43%; 26/61) in species in tropical or subtropical habitats (Fig. 2) but was also described in 33% (15/46) of temperate zones species (Supplementary Table S1). Short or prolonged torpor bouts in hibernators have not been observed in species from polar regions (0/2; Fig. 2). Seasonality was more concentrated in certain clades than others and was the most common condition in the Carnivora (5/5 species), the Sciuridae (i.e., marmots and squirrels) (16/20), and the Primates (7/8), while rare in other non-sciurid rodents, bats, marsupials, and Afrotherians (i.e., tenrecs, golden moles, and elephant shrews) (Fig. 2). Both seasonality and non-seasonality were found to be equally likely to be the ancestral torpor condition (0.499 vs. 0.501, respectively, Fig. 2).

### Predictability of torpor use

We found that 41% (111/275) of all species display some degree of *flexibility* in torpor use, while *predictable* torpor use (i.e., all individuals of one species use torpor always in the same way) was only recorded for 8% (23/275) of all species [data deficient: 51% (141/275)]. Flexible torpor use was highest in tropical and subtropical species (44%; 93/213), and although it was also found in species in temperate and polar regions (29%; 18/62), many Northern hemisphere species have highly predictable torpor use where all individuals of a population use torpor in the same way (24% (15/62) vs. 4% (8/213) in tropical and subtropical areas; Supplementary Fig. S1). Phylogenetic patterns in predictability mirror those found in seasonality with the majority of predictable species consisting of the Carnivora, the Primates, and the Sciuridae (i.e., marmots and squirrels). There was also a much higher likelihood that the ancestral condition was flexible, that is, non-predictable (0.70), than predictable (0.30, Supplementary Fig. S1).

## Discussion

Our comprehensive review of mammalian torpor use shows that opportunistic, non-seasonal torpor is more common in tropical and subtropical than in polar and temperate zone mammals, but it is not limited to these climatic zones and can also be found in species living in seasonal Northern hemisphere habitats with cold winters. Thus, the biogeographic distribution data support our hypothesis that non-seasonal and flexible use of torpor is likely the ancestral condition in mammals and that predictable seasonal torpor use is the derived form. However, the ancestral state reconstruction analyses were more ambiguous. Although we found a 70% likelihood for non-predictable, flexible torpor use to be the ancestral condition, there was an equal likelihood for seasonal and non-seasonal torpor. Similarly, the ancestral state analysis revealed an almost equal likelihood of any of the three torpor types being the ancestral condition. Furthermore, our prediction that seasonal torpor expression requiring extensive preparation would be restricted to cold, highly seasonal environments and only a few taxa was not supported.

These ambiguous results may be largely due to sampling bias, as much work has focused on studying torpor in species living in harsh and seasonal environments and seldom includes milder habitats. Geographical and phylogenetic biases in physiological datasets are unfortunately common and can severely limit both our ability to understand current patterns as well as to predict future outcomes (White et al. 2021). For example, strictly seasonal torpor and hibernation that requires preparation was mostly found in temperate and polar habitats (and there mostly concentrated in the Marmotini) but was also observed in about 10% of subtropical and tropical species. However, these were mainly clustered in one family of Malagasy lemurs, the Cheirogalidae (reviewed in Dausmann and Warnecke 2016; Nowack et al. 2020), which are known to use hibernation, live in the highly seasonal dry forest or montane regions of Madagascar. Comparable data are lacking from closely related species in less seasonal environments such as the eastern rainforests of Madagascar. We also found that for many species, information was missing about the possible occurrence of torpor outside of the dry and/or cold season. Thus, non-seasonal use of torpor may be more widespread than captured in our current dataset.

In contrast to our hypothesis, strictly seasonal torpor and hibernation that required preparation was not found to be restricted to highly seasonal temperate and polar zone habitats but also found in subtropical and tropical species. Despite strictly seasonal use by some species, torpor use can often still be relatively flexible, as torpor use may differ between individuals within a single population (reviewed in McKechnie and Mzilikazi 2011; Nowack et al. 2020). In the Malagasy mouse lemurs *Microcebus griseorufus* and *M. murinus*, for example, all individuals enter torpor during winter, but the extent varies depending on body condition, and individuals of one population can use short torpor, prolonged torpor, or hibernation in response to the same environmental conditions (Kobbe et al. 2011; Vuarin et al. 2013). Unpredictable use of torpor has also been found in Northern hemisphere species. In many species, such as the little brown bat *Myotis lucifugus*, the extent of torpor use varies in relation to the individual’s reproductive status, with pregnant individuals typically using shorter and shallower torpor bouts (Dzal and Brigham 2013), and torpor use in Mongolian hamsters *Meriones unguiculatus* in the laboratory was only found in females, but not in males (Watanabe et al. 2016). In contrast to subtropical species, a high percentage of Northern hemisphere species does, however, use torpor in a highly predictable manner, and all individuals of a population use torpor on the same days and in the same way. Our ancestral state analysis on the predictability of torpor use suggests that predictable torpor use may be a derived condition in response to highly seasonal habitats.

It is important to note that there may be a discrepancy between the species’ capacity to use non-seasonal torpor and their actual implementation of it, as the latter will reflect environmental conditions and not physiological capabilities (Landry-Cuerrier et al. 2008). Laboratory experiments have, for example, shown that even species known for their highly seasonal torpor use, such as thirteen-lined ground squirrels *Ictidomys tridecemlineatus*, can, if kept under artificial warm room conditions, change their torpor pattern from hibernation to regular short torpor bouts employed during winter, spring, and even summer (MacCannell and Staples 2021). Furthermore, although hibernation typically requires extensive preparation, the examples of the few exclusively subtropical and tropical species that do not show seasonal fattening or food hoarding suggest that under certain environmental conditions, a more flexible, non-seasonal use of hibernation may be possible. Nevertheless, our macroanalysis shows that opportunistic non-seasonal torpor use was found globally, and species known for their use of seasonal hibernation in winter can undergo shorter opportunistic bouts in summer, such as the European common garden dormice *Muscardinus avellanarius* (Pretzlaff et al. 2014), edible dormice *Glis glis* (Wilz and Heldmaier 2000; Hoelzl et al. 2015), hispid pocket mice *Chaetodipus (Perognatus) hispidus* (Wang and Hudson 1970), and a number of bat species (Fjelldal et al. 2022). For example, although brown long-eared bats *Plecotus auritus* use classical hibernation with long periods of torpor throughout winter, they also display variable patterns of torpor throughout the rest of the year, including short and prolonged torpor (Fjelldal et al. 2022). Further, although listed as temperate zone species here due to their mid-range distribution, the study on long-eared bats was undertaken in the northernmost part of the species’ range in the subarctic polar region, revealing that even in highly seasonal habitats, opportunistic torpor use throughout the year is widespread. Most hibernators were found to undergo significant phenotypic changes necessary to allow survival during long, cold, dry, and unproductive periods.

### Considerations about the evolution of endothermy

Importantly, our data provide further empirical support that flexible and opportunistic torpor use may be the ancestral form that would have been used by early mammals during the transition to whole-body endothermy (Grigg et al. 2004; Lovegrove 2012). Early mammals were likely small, nocturnal, and insectivorous, with low thermogenic capacity and high rates of heat loss (Crompton et al. 1978; O'Leary et al. 2013). Although the climatic conditions at this time would have been warmer than today, animals would still have to deal with conditions similar to today’s tropical and subtropical habitats, which can also see periods of low nighttime temperatures and varying food availability (Geiser et al. 2017). Our current understanding of the evolution of endothermy is that early mammals and birds evolved endothermy via a stepwise increase in metabolic rate that would have at least allowed individuals to decouple daytime activity from environmental conditions until insulation capacities increased (Crompton et al. 1978; Ruben 1995; Geiser et al. 2017), and it has been suggested that the process was facilitated by the use of opportunistic torpor during colder days (Geiser et al. 2017). More derived forms of torpor, including the highly seasonal and prolonged forms, would have then evolved during the periods of global cooling following the Eocene Climatic Optimum (∼40 Mya) concurrent with the extensive mammalian radiations following the K-Pg mass extinction event (Lovegrove 2012; Lovegrove 2017).

Interestingly, no single torpor use type came out as most likely to be the ancestral state with daily torpor having only a slightly higher likelihood than both prolonged torpor and hibernation. Earlier attempts to identify the ancestral torpor type included investigations of torpor use in clades that split from the rest of the mammal phylogeny at an earlier date, such as the Patagonian opossum (*Lestodelphys halli*, Order: Didelphimorphia), which was found to employ both short and deep prolonged bouts of torpor (Geiser and Martin 2013). This may suggest that such flexible use may be the torpor expression from which the more derived and seasonal forms of daily torpor and hibernation originated in marsupials. A similarly flexible torpor use with short and prolonged bouts of torpor and high seasonality has also been found in the monito del monte (*Dromiciops gliroides*, Order: Microbiotheria), a clade that sits between the South American and Australasian marsupials (Mejías et al. 2022). However, it needs to be noted that both species show tail fattening, which is likely a derived adaptation to seasonal habitats. In total, at least 38% of hibernators were found to be able to use short and/or prolonged torpor bouts. This percentage is, however, likely an underestimation, due to the high number of species for which data are lacking or which were only observed during one season, and the number of species employing multiple torpor use types is likely to increase with further study.

Interestingly, even species such as Djungarian hamsters that enter regular daily bouts of spontaneous torpor in winter after undergoing photoperiod-induced morphological changes can undergo opportunistic torpor in response to fasting throughout the year (Diedrich and Steinlechner 2012; Przybylska-Piech and Jefimow 2022). Such induced torpor bouts have been found to be shorter and less deep than seasonal torpor in winter (Diedrich and Steinlechner 2012) and are thus seen as a form of emergency response due to depleted energy reserves. In recent years, more and more incidents of torpor use as a form of emergency shutdown have been reported, and in many of these species, torpor is only used by a few individuals of the population (Jones and Geiser 1992; Christian and Geiser 2007; Barker et al. 2012; Nowack et al. 2013; Nowack et al. 2015; Dausmann et al. 2022; Nowack and Turbill 2022). Some of these species also show surprisingly low rewarming rates and relatively shallow decreases in body temperature (Barker et al. 2012; Nowack et al. 2013; Nowack and Turbill 2022), which does not support the view of torpor as a highly controlled mechanism where body temperature is reduced considerably and entry and rewarming from torpor are fast. The low rewarming rates have been attributed to depleted energy reserves (Nowack et al. 2013; Nowack and Turbill 2022). These less controlled torpor bouts in response to acute energetic bottlenecks may be very similar to torpor patterns in early mammaliaforms with individuals likely having relied on basking to rewarm from torpor as their thermogenic capacities would have been low (Geiser et al. 2017). Similar patterns are observed during the development of endothermy in juvenile mammals. At first, they are unable to maintain a high body temperature during nightly cold exposure, but then gradually increase thermal capacity, which enables them to maintain homeothermy for the first half of the night before they allow their body temperature to drop, but initially can only rewarm with the help of an external heat source (Hill 1976; Wacker et al. 2017). Basking to rewarm from torpor is also commonly found in extant adult mammals (e.g., Geiser et al. 2002; Mzilikazi et al. 2002; Geiser and Pavey 2007; Warnecke et al. 2008; Warnecke and Geiser 2010; Geiser et al. 2016; Wacker et al. 2017), and animals are able to crawl into the sun with body temperatures as low as 14.6°C degrees (Warnecke et al. 2008).

## Conclusion

Despite the increasing number of species found to use torpor in regions outside of the cold, seasonal temperate, and polar zones and the acknowledgement that torpor use is not primarily employed for winter survival, the classical Holarctic torpor pattern is still widely seen as a mammalian norm. This is perhaps partly due to a lack of consensus in the definitions of different patterns of torpor use, suggesting the need for more universally applied definitions of torpor such as those presented in our glossary. Our dataset revealed that there are a large number of species known to use torpor but are lacking details on many aspects of torpor use, including seasonality, predictability, and flexibility. Furthermore, our analyses were restricted to species that have been found to use torpor, which is in turn biased by geographical location, level of seasonality, and taxonomy. The vast majority of studies have examined torpor in species suspected to use it. Based on our findings as well as a recent synthesis of the literature, it is more likely that homeothermy is the exception and heterothermy (including torpor use) is the norm, at least in small mammals. We predict that as more data are obtained from free-ranging species, we are likely to see more instances of opportunistic torpor as well as potentially more types of torpor. In addition to highlighting the need to undertake more research on the seasonality and flexibility of torpor use, our data emphasize that the torpor patterns observed in the tropics and subtropics can no longer be considered exceptions to the hibernation rules derived from the Holarctic. Our findings further suggest that the non-seasonal, opportunistic, and flexible use of torpor is likely the ancestral state and that the seasonal, inflexible use of torpor is a derived form of heterothermy.

## Supplementary Material

icad067_Supplemental_File

## Data Availability

The data required to reproduce the above findings are available to download from: 10.6084/m9.figshare.23310731

## Notes

From the symposium “Daily torpor across birds and mammals: Recent progress and how do we advance the field” presented at the annual meeting of the Society for Integrative and Comparative Biology, January 3–7, 2023.

## References

[bib1] Aschoff J . 1963. Comparative physiology: diurnal rhythms. Annu Rev Physiol. 25:581–600. 13965146 10.1146/annurev.ph.25.030163.003053

[bib2] Barker JM , Cooper CE, Withers PC, Cruz-Neto AP. 2012. Thermoregulation by an Australian murine rodent, the ash-grey mouse (*Pseudomys albocinereus*). Comp Biochem Physiol A Mol Integr Physiol. 163:336–42. 22871479 10.1016/j.cbpa.2012.07.011

[bib3] Barnes BM . 1989. Freeze avoidance in a mammal: body temperatures below 0°C in an arctic hibernator. Science. 244:1593–5. 2740905 10.1126/science.2740905

[bib4] Bennett A , Ruben J. 1979. Endothermy and activity in vertebrates. Science. 206:649–54. 493968 10.1126/science.493968

[bib5] Boursot P , Din W, Anand R, Darviche D, Dod B, Von Deimling F, Talwar GP, Bonhomme F. 1996. Origin and radiation of the house mouse: mitochondrial DNA phylogeny. J Evolution Biol. 9:391–415.

[bib6] Boyles JG , Thompson AB, McKechnie AE, Malan E, Humphries MM, Careau V. 2013. A global heterothermic continuum in mammals. Global Ecol Biogeogr. 22:1029–39.

[bib7] Christian N , Geiser F. 2007. To use or not to use torpor? Activity and body temperature as predictors. Naturwissenschaften. 94:483–7. 17252241 10.1007/s00114-007-0215-5

[bib8] Cooper CE , Withers PC. 2004. Patterns of body temperature variation and torpor in the numbat, *Myrmecobius fasciatus* (Marsupialia: myrmecobiidae).J Therm Biol. 29:277–84.

[bib9] Crompton AW , Taylor CR, Jagger JA. 1978. Evolution of homeothermy in mammals. Nature. 272:333–6. 634356 10.1038/272333a0

[bib10] Dausmann KH , Glos J, Ganzhorn JU, Heldmaier G. 2004. Physiology: hibernation in a tropical primate. Nature. 429:825–6. 15215852 10.1038/429825a

[bib11] Dausmann KH , Körtner G, Aharon-Rotman Y, Currie S, Geiser F. 2022. Flexible employment of torpor in squirrel gliders (*Petaurus norfolcensis*): an adaptation to unpredictable climate?. Physiol Biochem Zool. 96:62–74. 36626839 10.1086/722131

[bib12] Dausmann KH , Levesque DL, Wein J, Nowack J. 2020. Ambient temperature cycles affect daily torpor and hibernation patterns in Malagasy tenrecs. Front Physiol. 11:522. 32547412 10.3389/fphys.2020.00522 PMC7270353

[bib13] Dausmann KH , Warnecke L. 2016. Primate torpor expression: ghost of the climatic past. Physiology. 31:398–408. 27708046 10.1152/physiol.00050.2015

[bib14] Diedrich V , Steinlechner S. 2012. Spontaneous daily torpor versus fasting-induced torpor in the Djungarian hamster (*Phodopus sungorus*): two sdes of a medal or distinct phenomena?In: Ruf T, Bieber C, Arnold W, Millesi E, editors. Living in a seasonal world. Berlin Heidelberg:Springer. p.231–42.

[bib15] Dzal YA , Brigham RM. 2013. The tradeoff between torpor use and reproduction in little brown bats (*Myotislucifugus*). J Comp Physiol B. 183:279–88. 22972361 10.1007/s00360-012-0705-4

[bib16] Fjelldal MA , Sørås R, Stawski C. 2022. Universality of torpor expression in bats. Physiol Biochem Zool. 95:326–39. 35622440 10.1086/720273

[bib17] French A . 1985. Allometries of the durations of torpid and euthermic intervals during mammalian hibernation: a test of the theory of metabolic control of the timing of changes in body temperature. J Comp Physiol B. 156:13–9. 3836228 10.1007/BF00692921

[bib21] Geiser F , Gasch K, Bieber C, Stalder GL, Gerritsmann H, Ruf T. 2016. Basking hamsters reduce resting metabolism, body temperature and energy costs during rewarming from torpor. J Exp Biol. 219:2166–72. 27207637 10.1242/jeb.137828

[bib22] Geiser F , Goodship N, Pavey CR. 2002. Was basking important in the evolution of mammalian endothermy?. Naturwissenschaften. 89:412–4. 12435094 10.1007/s00114-002-0349-4

[bib23] Geiser F , Körtner G. 2010. Hibernation and daily torpor in Australian mammals. Aust Zool. 35:204–15.

[bib24] Geiser F , Martin G. 2013. Torpor in the Patagonian opossum (*Lestodelphys halli*): implications for the evolution of daily torpor and hibernation. Naturwissenschaften. 100:975–81. 24045765 10.1007/s00114-013-1098-2

[bib25] Geiser F , Pavey CR. 2007. Basking and torpor in a rock-dwelling desert marsupial: survival strategies in a resource-poor environment. J Comp Physiol B. 177:885–92. 17674010 10.1007/s00360-007-0186-z

[bib27] Geiser F , Stawski C, Wacker CB, Nowack J. 2017. Phoenix from the ashes: fire, torpor, and the evolution of mammalian endothermy. Front Physiol. 8:842. 29163191 10.3389/fphys.2017.00842 PMC5673639

[bib18] Geiser F . 2004. Metabolic rate and body temperature reduction during hibernation and daily torpor. Annu Rev Physiol. 66:239–74. 14977403 10.1146/annurev.physiol.66.032102.115105

[bib19] Geiser F . 2020. Seasonal expression of avian and mammalian daily torpor and hibernation: not a simple summer-winter affair. Front Physiol. 11:436. 32508673 10.3389/fphys.2020.00436 PMC7251182

[bib20] Geiser F . 2021. Ecological physiology of daily torpor and hibernation. Cham, Switzerland: Springer.

[bib28] Grigg GC , Beard LA, Augee ML. 2004. The evolution of endothermy and its diversity in mammals and birds. Physiol Biochem Zool. 77:982–97. 15674771 10.1086/425188

[bib29] Grimpo K , Legler K, Heldmaier G, Exner C. 2013. That’s hot: golden spiny mice display torpor even at high ambient temperatures. J Comp Physiol B. 183:567–81. 23212435 10.1007/s00360-012-0721-4

[bib30] Gyhrs C , Macedo T, Bastos B, Salgado-Irazabal X, Hammadi M, Bouarakia O, Boratyński Z. 2022. High level of daily heterothermy in desert gerbils. J Trop Ecol. 38:451–3.

[bib31] Hill RW . 1976.The ontogeny of homeothermy in neonatal *Peromyscus leucopus*. Physiol Zool. 49:292–306.

[bib32] Hoelzl F , Bieber C, Cornils JS, Gerritsmann H, Stalder GL, Walzer C, Ruf T. 2015. How to spend the summer? Free-living dormice (*Glis glis*) can hibernate for 11 months in non-reproductive years. J Comp Physiol B. 185:931–9. 26293446 10.1007/s00360-015-0929-1 PMC4628641

[bib33] Hoffmann K . 1973. The influence of photoperiod and melatonin on testis size, body weight, and pelage colour in the Djungarian hamster (*Phodopus sungorus*). J Comp Physiol. 85:267–82.

[bib34] IUCN . 2022. The IUCN Red List of threatened species. Version 2022-2. [Internet]. Available from: https://www.iucnredlist.org (6 March 2023, date last accessed).

[bib35] Jones CJ , Geiser F. 1992. Prolonged and daily torpor in the feathertail glider, *Acrobates pygmaeus* (Marsupialia: acrobatidae). J Zool. 227:101–8.

[bib36] Jones KE , Bielby J, Cardillo M, Fritz SA, Odell J, Orme CDL, Safi K, Sechrest W, Boakes EH, Carbone C et al. 2009. PanTHERIA: a species-level database of life history, ecology, and geography of extant and recently extinct mammals. Ecology. 90:2648.

[bib37] Kobbe S , Ganzhorn J, Dausmann KH. 2011.Extreme individual flexibility of heterothermy in free-ranging Malagasy mouse lemurs (*Microcebus griseorufus*). J Comp Physiol B. 181:165–73. 20717683 10.1007/s00360-010-0507-5

[bib38] Landry-Cuerrier M , Munro D, Thomas DW, Humphries MM. 2008. Climate and resource determinants of fundamental and realized metabolic niches of hibernating chipmunks. Ecology. 89:3306–16. 19137938 10.1890/08-0121.1

[bib39] Levesque DL , Breit AM, Brown E, Nowack J, Welman S. 2023. Non-torpid heterothermy in mammals: another point along the homeothermy-hibernation continuum. Integr Comp Biol. ICB-2023-0027. 10.1093/icb/icad094 37407285

[bib40] Levesque DL , Nowack J, Stawski C. 2016. Modelling mammalian energetics: the heterothermy problem. Clim Chang Responses. 3:7.

[bib43] Lovegrove BG , Lobban KD, Levesque DL. 2014. Mammal survival at the Cretaceous–Palaeogene boundary: metabolic homeostasis In prolonged tropical hibernation in tenrecs. Proc Biol Sci. 281:20141304. 25339721 10.1098/rspb.2014.1304 PMC4213634

[bib41] Lovegrove BG . 2012. The evolution of endothermy in cenozoic mammals: a plesiomorphic-apomorphic continuum. Biol Rev. 87:128–62. 21682837 10.1111/j.1469-185X.2011.00188.x

[bib42] Lovegrove BG . 2017. A phenology of the evolution of endothermy in birds and mammals. Biol Rev. 92:1213–40. 27154039 10.1111/brv.12280

[bib44] Lyman CP , Willis JS, Malan A, Wang L. 1982. Hibernation and torpor in mammals and birds. New York (NY): Academic Press, Inc.

[bib45] MacCannell ADV , Staples JF. 2021. Elevated ambient temperature accelerates aspects of torpor phenology in an obligate hibernator. J Therm Biol. 96:102839. 33627277 10.1016/j.jtherbio.2021.102839

[bib46] McKechnie AE , Mzilikazi N. 2011. Heterothermy in afrotropical mammals and birds: a review. Integr Comp Biol. 51:349–63. 21705792 10.1093/icb/icr035

[bib47] Mejías C , Navedo JG, Sabat P, Franco LM, Bozinovic F, Nespolo RF. 2022. Body composition and energy savings by hibernation: lessons from the South American marsupial *Dromiciops gliroides*. Physiol Biochem Zool. 95:239–50. 35443149 10.1086/719932

[bib48] Mzilikazi N , Lovegrove BG, Ribble GO. 2002. Exogenous passive heating during torpor arousal in free-ranging rock elephant shrews, *Elephantulus myurus*. Oecologia. 133:307–14. 28466221 10.1007/s00442-002-1052-z

[bib49] Nowack J , Levesque DL, Reher S, Dausmann KH. 2020. Variable climates lead to varying phenotypes: “weird” mammalian torpor and lessons from non-Holarctic species. Front Ecol Evol. 8:60.

[bib50] Nowack J , Mzilikazi N, Dausmann KH. 2013. Torpor as an emergency solution in *Galago moholi*: heterothermy is triggered by different constraints. J Comp Physiol B. 183:547–56. 23242128 10.1007/s00360-012-0725-0

[bib51] Nowack J , Mzilikazi N, Dausmann KH. 2023. Saving energy via short and shallow torpor bouts. J Therm Biol. 114:103572. 37344030 10.1016/j.jtherbio.2023.103572

[bib52] Nowack J , Rojas AD, Körtner G, Geiser F. 2015. Snoozing through the storm: torpor use during a natural disaster. Sci Rep. 5:11243. 26073747 10.1038/srep11243 PMC4466894

[bib53] Nowack J , Stawski C, Geiser F. 2017. More functions of torpor and their roles in a changing world. J Comp Physiol B. 187:889–97. 28432393 10.1007/s00360-017-1100-y PMC5486538

[bib54] Nowack J , Turbill C. 2022. Survivable hypothermia or torpor in a wild-living rat: rare insights broaden our understanding of endothermic physiology. J Comp Physiol B. 192:183–92. 34668054 10.1007/s00360-021-01416-3 PMC8817056

[bib55] O'Leary MA , Bloch JI, Flynn JJ, Gaudin TJ, Giallombardo A, Giannini NP, Goldberg SL, Kraatz BP, Luo Z-X, Meng J et al. 2013. The placental mammal ancestor and the post–K-pg radiation of placentals. Science. 339:662–7. 23393258 10.1126/science.1229237

[bib56] Pagel M . 1994. Detecting correlated evolution on phylogenies: a general method for the comparative analysis of discrete characters. Proc R Soc Lond B. 255:37–45.

[bib57] Paradis E , Schliep K. 2019. Ape 5.0: an environment for modern phylogenetics and evolutionary analyses in R. Bioinformatics. 35:526–8. 30016406 10.1093/bioinformatics/bty633

[bib58] Pebesma E . 2018. Simple features for r: standardized support for spatial vector data. R J. 10:439–46.

[bib59] Pretzlaff I , Rau D, Dausmann KH. 2014. Energy expenditure increases during the active season in the small, free-living hibernator *Muscardinus avellanarius*. Mamm Biol. 79:208–14.

[bib60] Przybylska-Piech AS , Jefimow M. 2022. Siberian hamsters nonresponding to short photoperiod use fasting-induced torpor. J Exp Biol. 225:jeb244222. 10.1242/jeb.244222 35615921

[bib61] Reher S , Dausmann KH. 2021. Tropical bats counter heat by combining torpor with adaptive hyperthermia. Proc R Soc B. 288:20202059. 10.1098/rspb.2020.2059 PMC7892405 33434466

[bib62] Reher S , Ehlers J, Rabarison H, Dausmann KH. 2018. Short and hyperthermic torpor responses in the Malagasy bat *Macronycteris commersoni* reveal a broader hypometabolic scope in heterotherms. J Comp Physiol B. 188:1015–27. 30121696 10.1007/s00360-018-1171-4

[bib63] Ruben J . 1995. The evolution of endothermy in mammals and birds: from physiology to fossils. Annu Rev Physiol. 57:69–95. 7778882 10.1146/annurev.ph.57.030195.000441

[bib64] Ruf T , Gasch K, Stalder G, Gerritsmann H, Giroud S. 2021. An hourglass mechanism controls torpor bout length in hibernating garden dormice. J Exp Biol. 224:jeb243456. 34762135 10.1242/jeb.243456 PMC8714077

[bib65] Ruf T , Geiser F. 2015. Daily torpor and hibernation in birds and mammals. Biol Rev. 90:891–926. 25123049 10.1111/brv.12137 PMC4351926

[bib66] Shankar A , Cisneros INH, Thompson S, Graham CH, Powers DR. 2022. A heterothermic spectrum in hummingbirds. J Exp Biol. 225:jeb243208. 34989393 10.1242/jeb.243208

[bib67] Sheriff MJ , Fridinger RW, Tøien Ø, Barnes BM, Buck CL. 2013. Metabolic rate and prehibernation fattening in free-living Arctic ground squirrels. Physiol Biochem Zool. 86:515–27. 23995482 10.1086/673092

[bib68] Tattersall G . 2012. Diurnal changes in metabolic rate in pygmy marmosets: implications for sleep, torpor, and basal metabolism in primates. In: Ruf T, Bieber C, Arnold W, Millesi E, editors. Living in a seasonal world. Berlin Heidelberg: Springer Verlag. p. 471–80.

[bib69] Turner JM , Körtner G, Warnecke L, Geiser F. 2012. Summer and winter torpor use by a free-ranging marsupial. Comp Biochem Physiol A Mol Integr Physiol. 162:274–80. 22487484 10.1016/j.cbpa.2012.03.017

[bib70] Twente JW , Twente JA. 1964. An hypothesis concerning the evolution of heterothermy in bats. Annales Academiae Scientiarum Fennicae. Series A. IV. Vol. 71. Suomalainen Tiedeakat. p. 433–42.

[bib71] Upham NS , Esselstyn JA, Jetz W. 2019. Inferring the mammal tree: species-level sets of phylogenies for questions in ecology, evolution, and conservation. PLoS Biol. 17:e3000494. 31800571 10.1371/journal.pbio.3000494 PMC6892540

[bib72] Vuarin P , Dammhahn M, Henry P-Y. 2013. Individual flexibility in energy saving: body size and condition constrain torpor use. Funct Ecol. 27:793–9.

[bib73] Wacker CB , McAllan BM, Körtner G, Geiser F. 2017. The role of basking in the development of endothermy and torpor in a marsupial. J Comp Physiol B. 187:1029–38. 28283794 10.1007/s00360-017-1060-2

[bib74] Wang LC-H , Hudson JW. 1970. Some physiological aspects of temperature regulation in the normothermic and torpid hispid pocket mouse, *Perognathus hispidus*. Comp Biochem Physiol. 32:275–93. 5417459 10.1016/0010-406x(70)90941-2

[bib75] Warnecke L , Geiser F. 2010. The energetics of basking behaviour and torpor in a small marsupial exposed to simulated natural conditions. J Comp Physiol B. 180:437–45. 19888581 10.1007/s00360-009-0417-6

[bib76] Warnecke L , Turner J, Geiser F. 2007. Torpor and basking in a small arid zone marsupial. Naturwissenschaften. 95:73–8. 17684718 10.1007/s00114-007-0293-4

[bib77] Watanabe D , Hatase M, Sakamoto S, Koshimoto C, Shinohara A, Morita T. 2016. Torpor capability in two gerbil species, *Meriones unguiculatus* and *Tatera indica*. Jpn J Environ Entomol Zool. 27:9–16.

[bib78] White CR , Marshall DJ, Chown SL, Clusella-Trullas S, Portugal SJ, Franklin CE, Seebacher F. 2021. Geographical bias in physiological data limits predictions of global change impacts. Funct Ecol. 35:1572–8.

[bib79] Wilz M , Heldmaier G. 2000. Comparison of hibernation, estivation and daily torpor in the edible dormouse, *Glis glis*. J Comp Physiol B. 170:511–21. 11128441 10.1007/s003600000129

