# Supplementary material for: Rare and Opportunistic Use of Torpor in Mammals—An Echo from the Past?
Source: Integr Comp Biol. 2023 Jun 16;63(5):1049–59. doi: 10.1093/icb/icad067 (PMC10714912; doi:10.1093/icb/icad067)
Supplement: icad067_Supplemental_File [file icad067_supplemental_file.docx]

**Supplementary material:** Figure S1 and Table S1


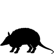

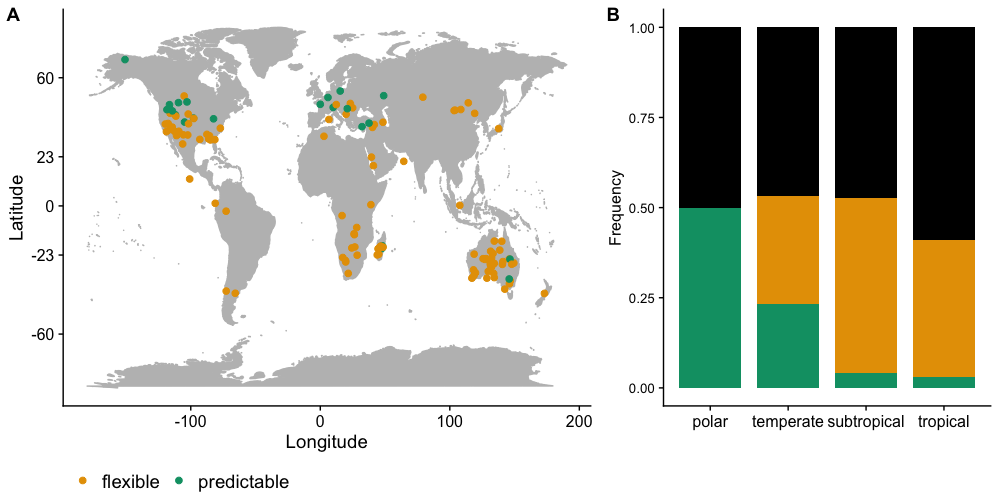


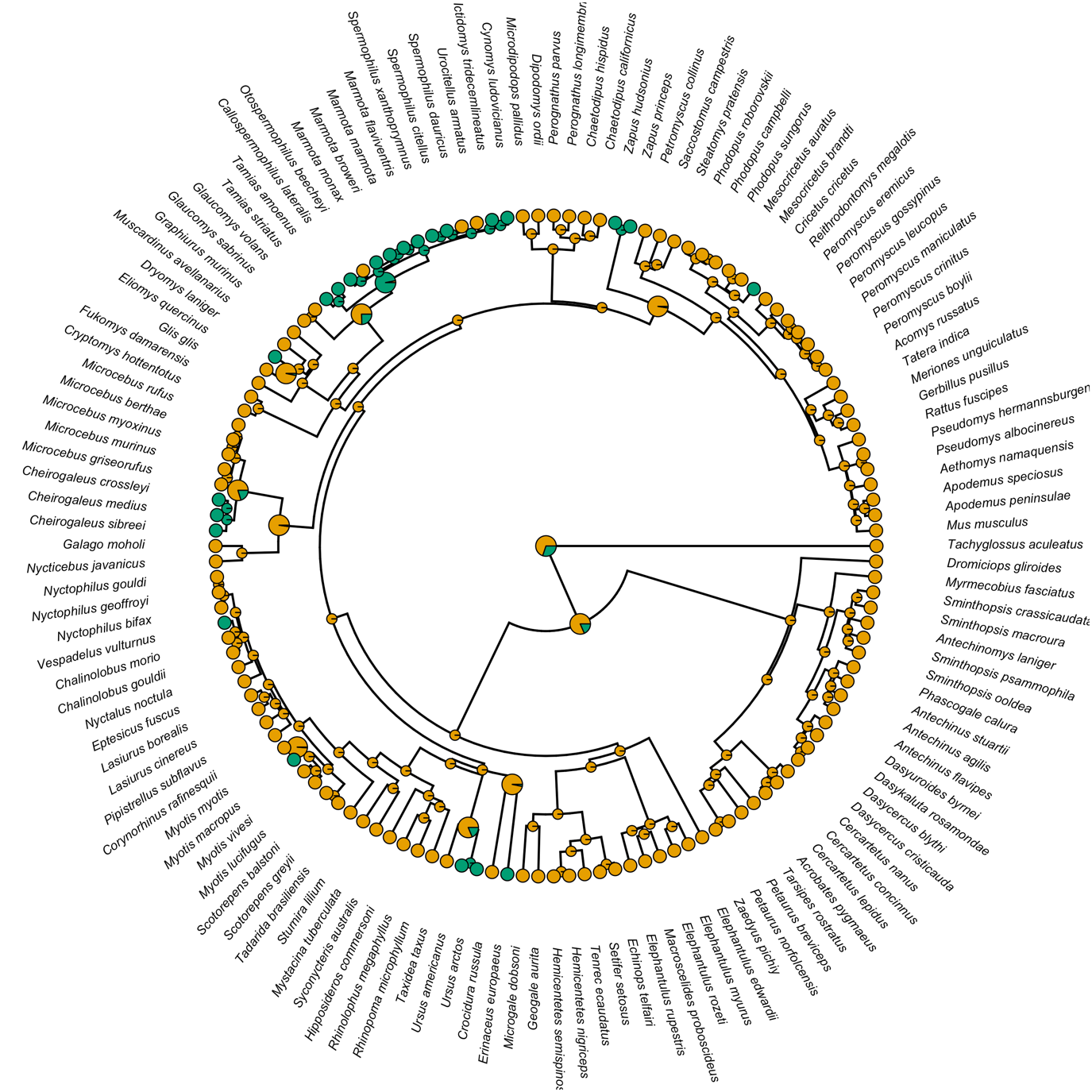


**C**

Rodentia

Chiroptera


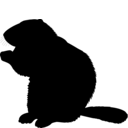

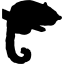

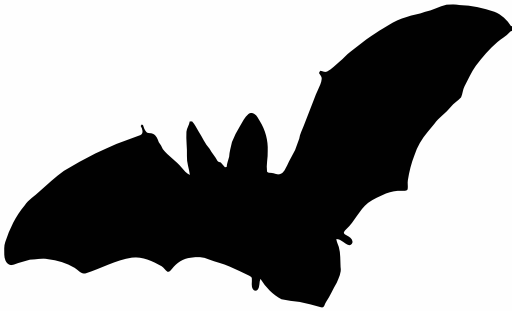

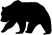

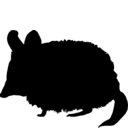

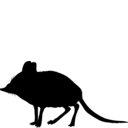

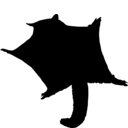


Primates


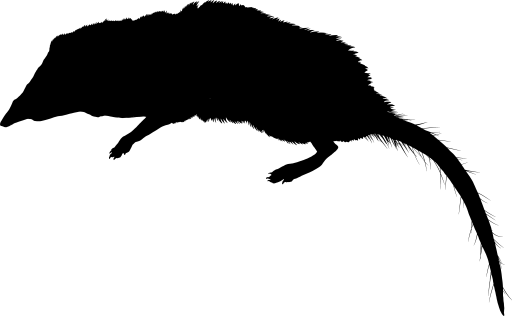

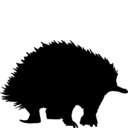

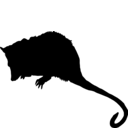


Dasyuromorpha

Afrotheria

**Figure S1:** **Phenotypic plasticity in torpor use.** A) Global distribution of plasticity torpor use. *Green:* predictable use, i.e. all individuals use torpor at the same time and undergo the same pattern of torpor; *Yellow*: flexible use of torpor, i.e. individuals differ in the timing and pattern of torpor use. B) Frequency distribution of phenotypic plasticity of torpor use based on climate zone. *Green*: predictable use; *Yellow*: flexible torpor use, *Black*: data deficient. Sample sizes for the four climate zones are: polar (2), temperate (60) subtropical (118), tropical (95). C) The phylogenetic relationships of species with data on phenotypic plasticity in torpor use. Pie charts at the nodes were estimated using ancestral state estimation and proportion of each colour represents the likelihood that the shared ancestor used torpor flexible (*yellow*) or predictable (*green*).

**Table S1:** List of heterothermic species used in the analysis, including: climatic region; torpor type based on the maximum torpor bout duration classed as either daily torpor (DT, longest duration <24h), prolonged torpor (PT, multiday bouts) or hibernation (HIB, series of multiday bouts); flexibility in torpor bout duration, e.g. whether a species that hibernates may also use short torpor bouts; seasonality of torpor use (yes/no), i.e. is torpor use restricted to one season (yes, i.e seasonal) such as the cold period or whether it is seen in different seasons (no, ie. non-seasonal use) and torpor predictability (yes/no), i.e. are all individuals using torpor in the same way (yes, i.e predictable) or do they differ in their likelihood to enter torpor or the pattern of torpor they are using. We also noted whether a hibernating species is known to prepare for hibernation (yes, i.e. food hoarding or fattening or no preparation (no)). Missing information was listed as data deficient (dd).

| **Species** | **Climate zone** | **Torpor**  **type** | **ST/PT** | **Seasonality** | **Torpor predictability** | **Hibernation preparation** | **Reference** |
| --- | --- | --- | --- | --- | --- | --- | --- |
| **MARSUPIALIA** |  |  |  |  |  |  |  |
| **Dasyuromorphia** |  |  |  |  |  |  |  |
| *Antechinomys laniger* | subtropical | DT | - | dd | no | - | (Geiser 1986) |
| *Antechinus agilis* | subtropical | DT | - | yes | no | - | (Wallis 1976) |
| *Antechinus flavipes* | subtropical | DT | - | no | no | - | (Geiser 1988; Stawski and Rojas 2016) |
| *Antechinus stuartii* | subtropical | DT | - | no | no | - | (Geiser 1988; Stawski et al. 2015; 2016) |
| *Antechinus swainsonii* | subtropical | DT | - | dd | dd | - | (Gotts 1976) |
| *Dasycercus blythi* | subtropical | DT | - | no | no | - | (Körtner et al. 2016) |
| *Dasycercus cristicauda* | subtropical | DT | - | no | no | - | (Geiser and Masters 1994; Körtner et al. 2008) |
| *Dasykaluta rosamondae* | tropical | DT | - | no | no | - | (Körtner et al. 2010) |
| *Dasyuroides byrnei* | subtropical | DT | - | no | no | - | (Geiser and Baudinette 1987; Körtner and Geiser 2011) |
| *Dasyurus geoffroii* | subtropical | DT | - | dd | dd | - | (Arnold 1976) |
| *Dasyurus hallucatus* | tropical | DT | - | dd | dd | - | (Cooper and Withers 2010) |
| *Dasyurus viverrinus* | temperate | DT | - | dd | dd | - | Moyle in (Reardon 1999) |
| *Myrmecobius fasciatus* | subtropical | DT | - | no | no | - | (Cooper and Withers 2004) |
| *Ningaui ridei* | tropical | DT | - | dd | dd | - | (Geiser and Cooper 2023) |
| *Ningaui yvonneae* | subtropical | DT | - | dd | dd | - | (Geiser and Baudinette 1987) |
| *Phascogale calura* | subtropical | DT | - | dd | no | - | (Pusey et al. 2013) |
| *Phascogale tapoatafa* | subtropical | DT | - | dd | dd | - | (Geiser and Cooper 2023) |
| *Planigale gilesi* | subtropical | DT | - | dd | dd | - | (Geiser and Baudinette 1988) |
| *Planigale ingrami* | tropical | DT | - | dd | dd | - | (Dawson and Wolfers 1978) |
| *Planigale maculata* | tropical | DT | - | dd | dd | - | (Morton and Lee 1978) |
| *Planigale tenuirostris* | subtropical | DT | - | dd | dd | - | (Dawson and Wolfers 1978) |
| *Pseudantechinus macdonnellensis* | tropical | DT | - | dd | dd | - | (Geiser and Pavey 2007) |
| *Sminthopsis crassicaudata* | subtropical | DT | - | no | no | - | (Geiser and Baudinette 1987; Warnecke et al. 2008) |
| *Sminthopsis dolichura* | subtropical | DT | - | dd | dd | - | (Geiser and Cooper 2023) |
| *Sminthopsis douglasi* | tropical | DT | - | dd | dd | - | (Muller 1996) |
| *Sminthopsis fuliginosus* | subtropical | DT | - | dd | dd | - | (Geiser and Cooper 2023) |
| *Sminthopsis granulipes* | subtropical | DT | - | dd | dd | - | (Geiser and Cooper 2023) |
| *Sminthopsis hirtipes* | subtropical | DT | - | dd | dd | - | (Geiser and Cooper 2023) |
| *Sminthopsis longicaudata* | subtropical | DT | - | dd | dd | - | (Burbidge et al. 2008) |
| *Sminthopsis macroura* | subtropical | DT | - | no | no | - | (Geiser and Baudinette 1987; Körtner and Geiser 2009) |
| *Sminthopsis murina* | subtropical | DT | - | yes | dd | - | (Geiser et al. 1984 ) |
| *Sminthopsis ooldea* | subtropical | DT | - | no | no | - | (Tomlinson et al. 2012) |
| *Sminthopsis psammophila* | subtropical | DT | - | dd | no | - | (Withers and Cooper 2009) |
| *Sminthopsis youngsoni* | tropical | DT | - | dd | dd | - | (Geiser and Cooper 2023) |
| **Didelpimorphia** |  |  |  |  |  |  |  |
| *Marmosa paraguayanus* | tropical | dd | dd | dd | dd | - | (Cooper et al. 2010) |
| *Gracilinanus agilis* | tropical | DT | - | dd | dd | - | (Cooper et al. 2009) |
| *Gracilinanus microtarsus* | subtropical | DT | - | dd | dd | - | (Morrison and McNab 1962) |
| *Lestodelphys halli* | subtropical | PT | yes | dd | dd | dd | (Geiser and Martin 2013) |
| *Marmosa robinsoni* | tropical | DT | - | dd | dd | - | (McNab 1978a) |
| *Monodelphis brevicaudata* | tropical | dd | dd | dd | dd | - | (McNab 1978b) |
| *Monodelphis domestica* | tropical | DT | - | dd | dd | - | (Busse et al. 2014) |
| *Thylamys elegans* | subtropical | DT | - | dd | dd | - | (Bozinovic et al. 2005; Opazo et al. 1999) |
| **Diprodontia** |  |  |  |  |  |  |  |
| *Acrobates pygmaeus* | subtropical | PT | dd | no | no | - | (Fleming 1985; Jones and Geiser 1992) |
| *Burramys parvus* | subtropical | HIB | dd | dd | dd | dd | (Geiser and Broome 1993) |
| *Cercartetus caudatus* | tropical | DT | - | dd | dd | - | (Atherton and Haffenden 1982) |
| *Cercartetus concinnus* | subtropical | PT | yes | no | no | - | (Turner and Geiser 2017; Turner et al. 2012b) |
| *Cercartetus lepidus* | subtropical | PT | yes | no | no | - | (Geiser 1987) |
| *Cercartetus nanus* | subtropical | HIB | yes | no | no | yes | (Turner et al. 2012a) |
| *Gymnobelideus leadbeateri* | subtropical | DT | - | dd | dd | - | (Smith 1980) |
| *Petaurus australis* | subtropical | dd | dd | dd | dd | dd | (Geiser and Körtner 2010) |
| *Petaurus breviceps* | tropical | DT | - | dd | no | - | (Christian and Geiser 2007; Nowack and Geiser 2016; Nowack et al. 2015) |
| *Petaurus norfolcensis* | subtropical | DT | - | dd | no | - | (Dausmann et al. 2023) |
| *Tarsipes rostratus* | subtropical | DT | - | dd | no | - | (Collins et al. 1987; Withers et al. 1989) |
| **Microbiotheria** |  |  |  |  |  |  |  |
| *Dromiciops gliroides* | subtropical | PT | yes | no | no | - | (Nespolo et al. 2021) |
| **Notoryctemorphia** |  |  |  |  |  |  |  |
| *Notoryctes caurinus* | subtropical | dd | Dd | dd | dd | dd | (Withers et al. 2000) |
| *Notoryctes typhlops* | tropical | dd | Dd | dd | dd | dd | (Tyndale-Biscoe 1973) |
| **Paucituberculata** |  |  |  |  |  |  |  |
| *Rhyncholestes raphanurus* | temperate | dd | dd | dd | dd | - | Kelt and Martinez (1989) |
| ***MONOTREMATA*** |  |  |  |  |  |  |  |
| *Tachyglossus aculeatus* | tropical | HIB | yes | no | no | yes | (Grigg and Beard 2000; Nowack et al. 2016) |
| ***PLACENTALIA*** |  |  |  |  |  |  |  |
| **Afrosoricida** |  |  |  |  |  |  |  |
| *Amblysomus hottentotus* | subtropical | PT | yes | dd | dd | dd | (Scantlebury et al. 2008) |
| *Echinops telfairi* | tropical | HIB | yes | no | no | no | (Dausmann et al. 2020; Lovegrove and Génin 2008; Wein 2010) |
| *Eremitalpa granti* | subtropical | dd | dd | dd | dd | dd | (Fielden et al. 1990; Seymour et al. 1998) |
| *Geogale aurita* | tropical | DT | - | no | no | - | (Stephenson and Racey 1993a) |
| *Hemicentetes nigriceps* | tropical | dd | dd | no | no | - | (Stephenson and Racey 1994) |
| *Hemicentetes semispinosus* | tropical | dd | dd | no | no | - | (Stephenson and Racey 1994) |
| *Microgale dobsoni* | tropical | dd | dd | dd | no | dd | (Stephenson and Racey 1993b) |
| *Microgale talazaci* | tropical | dd | dd | dd | dd | dd | (Stephenson and Racey 1993b) |
| *Neamblysomus julianae* | subtropical | DT | - | dd | dd | - | (Jackson et al. 2009) |
| *Setifer setosus* | tropical | HIB | yes | no | no | yes | (Levesque and Lovegrove 2014; Lovegrove et al. 2014a) |
| *Tenrec ecaudatus* | tropical | HIB | yes | no | no | yes | (Lovegrove et al. 2014b; Treat et al. 2018) |
| **Carnivora** |  |  |  |  |  |  |  |
| *Meles meles* | temperate | HIB | yes | yes | dd | - | (Fowler and Racey 1988; Tanaka 2006) |
| *Mephitis mephitis* | temperate | DT | - | yes | dd | - | (Hwang et al. 2007) |
| *Proteles cristata* | tropical | dd | dd | dd | dd | - | (Anderson 2004) |
| *Taxidea taxus* | subtropical | DT | - | yes | no | - | (Harlow 1981; Symes et al. 2019) |
| *Ursus americanus* | temperate | HIB | dd | yes | yes | yes | (Tøien et al. 2011) |
| *Ursus arctos* | temperate | HIB | dd | yes | yes | yes | (Hissa et al. 1994) |
| **Chiroptera** |  |  |  |  |  |  |  |
| *Barbastella barbastellus* | temperate | HIB | dd | dd | dd | dd | (Gottfried 2009; Pohl 1961) |
| *Carollia perspicillata* | tropical | DT | - | dd | dd | - | (Audet and Thomas 1997) |
| *Chalinolobus gouldii* | subtropical | HIB | dd | dd | no | dd | (Hosken and Withers 1997; Stawski and Currie 2016) |
| *Chalinolobus morio* | subtropical | HIB | yes | no | no | dd | (Turbill 2006) |
| *Corynorhinus rafinesquii* | subtropical | PT | yes | no | no | yes | (Johnson 2012) |
| *Dobsonia minor* | tropical | dd | dd | dd | dd | dd | (McNab and Bonaccorso 2001) |
| *Eptesicus fuscus* | subtropical | HIB | dd | no | no | yes | (Willis et al. 2005b) |
| *Glossophaga soricina* | tropical | DT | - | dd | dd | - | (Kelm and Helversen 2007) |
| *Hipposideros arminger* | tropical | HIB | yes | dd | dd | no | (Liu and Karasov 2011; 2012) |
| *Hipposideros commersoni* | tropical | HIB | yes | no | no | yes | (Reher and Dausmann 2021; Reher et al. 2018) |
| *Lasiurus borealis* | subtropical | HIB | dd | dd | no | yes | (Dunbar and Tomasi 2006) |
| *Lasiurus cinereus* | tropical | PT | yes | dd | no | - | (Cryan and Wolf 2003; Willis et al. 2006) |
| *Macroderma gigas* | tropical | dd | dd | dd | dd | dd | Unpublished in (Geiser and Stawski 2011) |
| *Macroglossus minimus* | tropical | DT | - | dd | dd | - | (Bartels et al. 1998) |
| *Megaderma lyra* | tropical | dd | dd | dd | dd | dd | (Kulzer 1965) |
| *Megaloglossus woermanni* | tropical | DT | - | dd | dd | - | (Kulzer and Storf 1980) |
| *Miniopterus natalensis* | tropical | HIB | dd | dd | dd | no | (Pretorius et al. 2021) |
| *Miniopterus schreibersii* | tropical | HIB | dd | yes | dd | no | (Hall 1982) |
| *Molossus molossus* | tropical | DT | - | dd | dd | - | (O'Mara et al. 2017) |
| *Mops condylurus* | tropical | DT | - | dd | dd | - | (Vivier and Van der Merwe 2007) |
| *Ozimops petersi (Mormopterus species 3)* | subtropical | PT | yes | no | dd | - | (Bondarenco et al. 2014) |
| *Myotis adversus* | tropical | HIB | dd | dd | dd | dd | (Kulzer et al. 1970) |
| *Myotis lucifugus* | temperate | HIB | yes | no | no | yes | (Dzal and Brigham 2013) |
| *Myotis macropus* | subtropical | PT | yes | dd | yes | - | (Barratt et al. 2022) |
| *Myotis myotis* | temperate | HIB | yes | no | no | yes | (Pohl 1961; Wojciechowski et al. 2007) |
| *Myotis nattereri* | temperate | HIB | dd | dd | dd | yes | (Hope and Jones 2012) |
| *Myotis thysanodes* | subtropical | HIB | yes | no | dd | yes | (Alston et al. 2022; O'Farrell and Studier 1970) |
| *Myotis velifer* | subtropical | HIB | dd | dd | dd | yes | (Riedesel and Williams 1976; Tinkle and Patterson 1965) |
| *Myotis vivesi* | subtropical | HIB | yes | no | no | no | (Salinas et al. 2014) |
| *Mystacina tuberculata* | temperate | PT | yes | dd | no | dd | (Czenze et al. 2017) |
| *Natalus tumidirostris* | tropical | dd | dd | dd | dd | dd | (Genoud et al. 1990) |
| *Nyctalus noctula* | subtropical | HIB | yes | no | no | yes | (Keicher et al. 2022) |
| *Nycteris thebaica* | tropical | DT | - | dd | dd | - | (Cory Toussaint and McKechnie 2012) |
| *Nyctimene albiventer* | tropical | DT | - | dd | dd | - | (Bartholomew et al. 1970) |
| *Nyctimene robinsoni* | tropical | dd | dd | dd | dd | dd | (Riek et al. 2010) |
| *Nyctophilus bifax* | tropical | PT | yes | no | no | no | (Stawski and Geiser 2010; 2012; Stawski et al. 2009) |
| *Nyctophilus geoffroyi* | subtropical | HIB | yes | no | no | no | (Geiser and Brigham 2000; Turbill and Geiser 2008) |
| *Nyctophilus gouldi* | subtropical | HIB | yes | no | no | yes | (Geiser and Brigham 2000; Turbill and Geiser 2008) |
| *Otonycteris hemprichii* | subtropical | DT | - | no | dd | - | (Daniel et al. 2010; Marom et al. 2006) |
| *Perimyotis subflavus* | subtropical | HIB | yes | no | no | yes | (Newman 2020) |
| *Peropteryx macrotis* | tropical | DT | - | dd | dd | - | (Genoud et al. 1990) |
| *Pipistrellus kuhlii* | subtropical | HIB | yes | dd | dd | dd | (Muñoz-Garcia et al. 2012) |
| *Pipistrellus pipistrellus* | temperate | HIB | yes | dd | dd | dd | (Genoud and Christe 2011; Kulzer 1965) |
| *Pipistrellus tenuipinnis* | tropical | PT | dd | dd | dd | dd | (Eisentraut 1956) |
| *Plecotus auritus* | temperate | HIB | yes | no | dd | dd | (Sørås et al. 2022) |
| *Rhinolophus ferrumequinum* | subtropical | HIB | dd | dd | dd | yes | (Kulzer 1965; Park et al. 2000) |
| *Rhinolophus hipposideros* | subtropical | HIB | dd | dd | dd | dd | (Harmata 1987) |
| *Rhinolophus megaphyllus* | tropical | dd | dd | no | no | dd | (Young 2001) |
| *Rhinonicteris aurantia* | tropical | dd | dd | dd | dd | dd | (Kulzer et al. 1970) |
| *Rhinopoma hardwickii* | tropical | HIB | dd | dd | dd | yes | (Levin et al. 2015) |
| *Rhinopoma microphyllum* | tropical | HIB | yes | no | no | yes | (Levin et al. 2012; Levin et al. 2015) |
| *Scotophilus dinganii* | tropical | DT | - | dd | dd | - | (Jacobs et al. 2007) |
| *Scotophilus mhlanganii* | tropical | DT | - | dd | dd | - | (Jacobs et al. 2007) |
| *Scotorepens balstoni* | subtropical | DT | - | dd | no | - | (Bondarenco et al. 2016) |
| *Scotorepens greyii* | tropical | DT | - | dd | no | - | (Bondarenco et al. 2016) |
| *Sturnira erythromos* | tropical | DT | - | dd | dd | - | (Soriano et al. 2002) |
| *Sturnira lilium* | tropical | DT | - | dd | no | - | (Audet and Thomas 1997) |
| *Syconycteris australis* | tropical | DT | - | no | no | - | (Coburn and Geiser 1998) |
| *Tadarida aegyptiaca* | tropical | PT | yes | dd | dd | - | (Cory Toussaint et al. 2010) |
| *Tadarida brasiliensis* | tropical | DT | - | dd | no | - | (Herreid and Schmidt-Nielsen 1966; Soriano et al. 2002) |
| *Tadarida teniotis* | subtropical | HIB | yes | dd | dd | dd | (Arlettaz et al. 2000; Marom et al. 2006) |
| *Taphozous australis* | tropical | dd | dd | dd | dd | dd | (Kulzer et al. 1970) |
| *Taphozous melanopogon* | tropical | dd | dd | dd | dd | dd | (Kulzer 1965) |
| *Vampyrops helleri* | tropical | dd | dd | dd | dd | dd | (Rasweiler 1973) |
| *Vespadelus vulturnus* | subtropical | HIB | yes | dd | yes | dd | (Willis et al. 2005a) |
| **Erinaceomorpha** |  |  |  |  |  |  |  |
| *Atelerix algirus* | subtropical | HIB | dd | dd | dd | dd | (Mouhoub-Sayah et al. 2008) |
| *Atelerix frontalis* | tropical | HIB | yes | dd | dd | yes | (Hallam and Mzilikazi 2011) |
| *Erinaceus amurensis* | subtropical | HIB | dd | dd | dd | dd | (Smith and Xie 2008) |
| *Erinaceus europaeus* | temperate | HIB | dd | yes | yes | yes | (Fowler and Racey 1990) |
| *Erinaceus roumanicus* | temperate | HIB | dd | yes | dd | dd | (Rutovskaya and Diatroptov 2022; Rutovskaya et al. 2019) |
| *Hemiechinus auritus* | subtropical | HIB | dd | dd | dd | dd | (Smith and Xie 2008) |
| *Mesechinus dauuricus* | temperate | HIB | dd | dd | dd | dd | (Smith and Xie 2008) |
| **Macroscelidea** |  |  |  |  |  |  |  |
| *Elephantulus edwardii* | subtropical | PT | yes | yes | no | no | (Geiser and Mzilikazi 2011) |
| *Elephantulus myurus* | tropical | PT | yes | no | no | no | (Lovegrove et al. 2001; Mzilikazi and Lovegrove 2004; Mzilikazi and Lovegrove 2005) |
| *Elephantulus rozeti* | subtropical | DT | no | dd | no | - | (Lovegrove et al. 2001) |
| *Elephantulus rupestris* | subtropical | DT | - | no | no | - | (Boyles et al. 2012b; Oelkrug et al. 2012) |
| *Macroscelides proboscideus* | subtropical | DT | - | dd | no | - | (Lovegrove et al. 1999) |
| **Primates** |  |  |  |  |  |  |  |
| *Allocebus trichotis* | tropical | DT | - | dd | dd | - | (Dausmann 2014) |
| *Cheirogaleus crossleyi* | tropical | HIB | dd | yes | yes | yes | (Blanco and Rahalinarivo 2010) |
| *Cheirogaleus major* | tropical | HIB | dd | dd | dd | dd | (Lahann 2007) |
| *Cheirogaleus medius* | tropical | HIB | dd | yes | yes | yes | (Dausmann et al. 2004; Lahann and Dausmann 2011) |
| *Cheirogaleus sibreei* | tropical | HIB | dd | yes | yes | yes | (Blanco et al. 2013) |
| *Galago moholi* | tropical | DT | - | yes | no | - | (Nowack et al. 2013) |
| *Lepilemur edwardsi* | tropical | dd | dd | dd | dd | dd | (Bethge et al. 2021) |
| *Loris tardigradus* | tropical | DT | - | dd | dd | - | Pers. Com. KAI Nekaris; (Müller et al. 1985) |
| *Microcebus berthae* | tropical | DT | - | yes | no | - | (Ortmann et al. 1997; Schmid et al. 2000)* published as M. myoxinus |
| *Microcebus griseorufus* | tropical | HIB | yes | yes | no | yes | (Kobbe et al. 2011) |
| *Microcebus lehilahytsara* | tropical | HIB | dd | yes | dd | yes | (Andriambeloson et al. 2020) |
| *Microcebus murinus* | tropical | HIB | yes | no | no | yes | (Giroud et al. 2008; Schmid and Speakman 2000) |
| *Microcebus myoxinus* | tropical | DT | - | dd | no | - | (Schmid et al. 2000) |
| *Microcebus ravelobensis* | tropical | DT | - | dd | dd | - | (Lovegrove et al. 2014a) |
| *Microcebus rufus* | tropical | PT | yes | dd | no | dd | (Atsalis 1999; Randrianambinina et al. 2003) |
| *Mirza coquereli* | tropical | DT | - | dd | dd | - | (Dausmann 2008) |
| *Nycticebus javanicus* | tropical | DT | - | dd | no | - | (Reinhard 2019) |
| *Nycticebus pygmaeus* | tropical | PT | yes | dd | dd | yes | (Ruf et al. 2015) |
| **Rodentia** |  |  |  |  |  |  |  |
| *Acomys russatus* | tropical | PT | yes | no | no | - | (Barak et al. 2018; Levy et al. 2011) |
| *Aethomys namaquensis (Micaelamys namaquensis)* | tropical | dd | dd | no | no | - | (Boyles et al. 2012a; Withers et al. 1980) |
| *Allactaga euphratica* | subtropical | HIB | dd | dd | dd | - | (Çolak and Yi̇ği 1998) |
| *Allactaga sibirica* | temperate | HIB | dd | dd | dd | dd | (Smith and Xie 2008) |
| *Allactaga williamsi* | subtropical | HIB | dd | dd | dd | dd | Çolak and Yi̇ği, 1998) |
| *Apodemus peninsulae* | temperate | DT | - | dd | no | - | (Masaki et al. 2005) |
| *Apodemus speciosus* | subtropical | DT | - | dd | no | - | (Eto et al. 2014) |
| *Baiomys taylori* | subtropical | DT | - | dd | dd | - | (Hudson 1965) |
| *Callospermophilus lateralis* | temperate | HIB | dd | dd | yes | yes | (Healy et al. 2012) |
| *Callospermophilus saturatus* | temperate | HIB | dd | yes | dd | yes | (Geiser et al. 1990) |
| *Calomys musculinus* | subtropical | DT | - | dd | dd | - | (Bozinovic and Rosenmann 1988) |
| *Calomys venustus* | subtropical | DT | - | dd | dd | - | (Caviedes-Vidal et al. 1990) |
| *Chaetodipus californicus* | subtropical | DT | - | no | no | - | (Tucker 1965) |
| *Chaetodipus hispidus* | subtropical | HIB | yes | dd | no | dd | (Wang and Hudson 1970) |
| *Cricetulus barabensis* | temperate | HIB | yes | dd | dd | dd | (Smith and Xie 2008) |
| *Cricetus cricetus* | temperate | HIB | yes | yes | yes | yes | (Siutz et al. 2018; Wassmer and Wollnik 1997) |
| *Cryptomys hottentotus* | tropical | DT | - | no | no | - | (Bennett et al. 1993) |
| *Cynomys leucurus* | temperate | HIB | dd | dd | dd | dd | (Bakko and Nahorniak 1986) |
| *Cynomys ludovicianus* | subtropical | HIB | dd | yes | yes | dd | (Lehmer et al. 2001) |
| *Cynomys parvidens* | subtropical | HIB | dd | dd | dd | dd | (Lehmer and Biggins 2005) |
| *Dryomys laniger* | subtropical | HIB | yes | yes | yes | yes | (Kart Gür et al. 2014) |
| *Dryomys nitedula* | tropical | HIB | dd | dd | dd | dd | (Nevo and Amir 1964) |
| *Dipodomys merriami* | Subtropical | DT | - | dd | dd | - | (Yousef and Dill 1971) |
| *Dipodomys ordii* | subtropical | DT | - | yes | no | - | (Gummer 2005) |
| *Dipus sagitta* | temperate | HIB | dd | dd | dd | dd | (Smith and Xie 2008) |
| *Eliomys quercinus* | temperate | HIB | yes | no | no | yes | (Daan 1973; Giroud et al. 2014) |
| *Fukomys damarensis* | tropical | dd | dd | yes | no | - | (Streicher 2010; Streicher et al. 2011) |
| *Gerbillus amoenus* | subtropical | DT | - | dd | dd | - | (Gyhrs et al. 2022) |
| *Gerbillus gerbillus* | tropical | DT | - | dd | dd | - | (Gyhrs et al. 2022) |
| *Gerbillus pusillus* | tropical | DT | - | dd | no | - | (Buffenstein and Jarvis 1985) |
| *Gerbillus species 1* | subtropical | DT | - | dd | dd | - | (Gyhrs et al. 2022) |
| *Glaucomys sabrinus* | temperate | DT | - | yes | no | - | (Olson et al. 2017) |
| *Glaucomys volans* | subtropical | DT | - | yes | no | - | (Muul 1968; Olson et al. 2017) |
| *Glirulus japonicus* | subtropical | HIB | dd | dd | dd | dd | (Otsu and Kimura 1993) |
| *Glis glis* | temperate | HIB | yes | no | no | yes | (Hoelzl et al. 2015; Wilz and Heldmaier 2000) |
| *Graphiurus murinus* | tropical | PT | yes | no | no | - | (Mzilikazi et al. 2012; Webb and Skinner 1996) |
| *Graphiurus ocularis* | subtropical | PT | dd | dd | dd | - | (Channing 1984; Perrin and Ridgard 1999) |
| *Ictidomys mexicanus* | subtropical | HIB | yes | dd | dd | dd | (Neumann and Cade 1965) |
| *Ictidomys tridecemlineatus* | temperate | HIB | dd | no | yes | yes | (Kisser and Goodwin 2012) |
| *Jaculus orientalis* | subtropical | HIB | yes | dd | dd | dd | (El Ouezzani et al. 2011) |
| *Marmota baibacina* | temperate | HIB | dd | dd | dd | dd | (Smith and Xie 2008) |
| *Marmota broweri* | polar | HIB | dd | yes | yes | yes | (Lee et al. 2009) |
| *Marmota caudata* | subtropical | HIB | dd | dd | dd | dd | (Smith and Xie 2008) |
| *Marmota flaviventris* | temperate | HIB | yes | yes | yes | yes | (Hill and Florant 2000) |
| *Marmota himalayana* | subtropical | HIB | dd | dd | dd | dd | (Smith and Xie 2008) |
| *Marmota marmota* | temperate | HIB | dd | yes | yes | yes | (Arnold et al. 2011) |
| *Marmota monax* | temperate | HIB | dd | yes | yes | yes | (Zervanos et al. 2013) |
| *Marmota sibirica* | temperate | HIB | dd | dd | dd | dd | (Smith and Xie 2008) |
| *Meriones unguiculatus* | temperate | DT | - | dd | no | - | (Watanabe et al. 2016) |
| *Mesocricetus auratus* | subtropical | HIB | dd | yes | no | yes | (Giroud et al. 2013; Pohl 1961; Terada and Ibuka 2000) |
| *Mesocricetus brandti* | subtropical | HIB | yes | dd | no | yes | (Batavia et al. 2013; Lyman et al. 1981) |
| *Microdipodops pallidus* | subtropical | HIB | yes | no | no | yes | (Bartholomew and MacMillen 1961; Brown and Bartholomew 1969; French 1989) |
| *Mus musculus* | subtropical | DT | - | no | no | - | (Renninger et al. 2020; Schubert et al. 2010) |
| *Muscardinus avellanarius* | temperate | HIB | yes | no | no | yes | (Pretzlaff and Dausmann 2012; Pretzlaff et al. 2014) |
| *Otospermophilus beecheyi* | subtropical | HIB | dd | no | no | dd | (Davis and Swade 1983; Strumwasser 1960) |
| *Otospermophilus variegatus* | subtropical | HIB | dd | yes | dd | yes | (Pengelley 1964; Pengelley and Kelly 1966) |
| *Perognathus longimembris* | subtropical | HIB | dd | no | no | no | (Bartholomew and Cade 1957) |
| *Perognathus parvus* | temperate | HIB | dd | yes | no | yes | (MacMillen 1983) |
| *Peromyscus boylii* | subtropical | DT | - | no | no | - | (Morhardt 1970) |
| *Peromyscus crinitus* | subtropical | DT | - | no | no | - | (Morhardt 1970) |
| *Peromyscus eremicus* | subtropical | DT | - | no | no | - | (MacMillen 1965; Morhardt 1970) |
| *Peromyscus gossypinus* | subtropical | DT | - | dd | no | - | (Tannenbaum and Pivorun 1984) |
| *Peromyscus leucopus* | subtropical | DT | - | no | no | - | (Tannenbaum and Pivorun 1984; 1988) |
| *Peromyscus maniculatus* | temperate | DT | - | no | no | - | (Geiser 1991; Tannenbaum and Pivorun 1988; 1989) |
| *Petromyscus collinus* | subtropical | DT | - | dd | no | - | (Withers et al. 1980) |
| *Phodopus campelli* | temperate | DT | - | dd | no | - | (Khrushchova et al. 2018) |
| *Phodopus roborovskii* | temperate | DT | - | dd | no | - | (Chi et al. 2016) |
| *Phodopus sungorus* | temperate | DT | - | no | no | - | (Haugg et al. 2021; Przybylska-Piech and Jefimow 2022; Ruf et al. 1993) |
| *Phyllotis darwini* | subtropical | DT | - | dd | dd | - | (Bozinovic and Marquet 1991) |
| *Pseudomys albocinereus* | subtropical | DT | - | dd | no | - | (Barker et al. 2012) |
| *Pseudomys hermannsburgensis* | subtropical | dd | - | dd | no | - | (Tomlinson et al. 2007) |
| *Rattus fuscipes* | subtropical | DT | - | dd | no | - | (Nowack and Turbill 2022) |
| *Reithrodontomys megalotis* | subtropical | DT | - | dd | no | - | (Thompson 1985) |
| *Saccostomus campestris* | tropical | DT | - | no | no | - | (Lovegrove and Raman 1998; Mzilikazi and Lovegrove 2002) |
| *Sicista betulina* | temperate | HIB | dd | dd | dd | dd | (Cade 1964) |
| *Spermophilus brevicauda* | temperate | HIB | dd | no | dd | dd | (Smith and Xie 2008) |
| *Spermophilus citellus* | temperate | HIB | dd | yes | yes | yes | (Németh et al. 2010) |
| *Spermophilus dauricus* | temperate | HIB | yes | dd | no | dd | (Yang et al. 2011) |
| *Spermophilus ralli* | temperate | HIB | dd | dd | dd | dd | (Smith and Xie 2008) |
| *Spermophilus undulatus* | temperate | HIB | dd | dd | dd | dd | (Smith and Xie 2008) |
| *Spermophilus xanthoprymnus* | subtropical | HIB | dd | yes | yes | yes | (Kart Gür et al. 2009) |
| *Steatomys pratensis* | tropical | DT | - | no | no | - | (Perrin and Richardson 2004) |
| *Tamias amoenus* | temperate | HIB | dd | yes | yes | yes | (Geiser et al. 1990) |
| *Tamias sibiricus* | temperate | HIB | dd | dd | dd | dd | (Kawamichi 1993; Smith and Xie 2008) |
| *Tamias striatus* | temperate | HIB | yes | yes | yes | yes | (Levesque and Tattersall 2010) |
| *Tatera indica* | tropical | DT | - | dd | no | - | (Watanabe et al. 2016) |
| *Urocitellus armatus* | temperate | HIB | dd | yes | no | dd | (Cranford 1986) |
| *Urocitellus beldingi* | temperate | HIB | dd | dd | dd | yes | (French 1985) |
| *Urocitellus columbianus* | temperate | HIB | dd | yes | dd | yes | (Young 1990) |
| *Urocitellus elegans* | temperate | HIB | dd | dd | dd | dd | (Harlow and Jr. 1986) |
| *Urocitellus parryii* | polar | HIB | dd | yes | dd | yes | (Frank et al. 2008; Williams et al. 2014) |
| *Urocitellus richardsonii* | temperate | HIB | dd | yes | dd | yes | (Hudson and Deavers 1973; Wang 1978) |
| *Xerospermophilus mohavensis* | subtropical | HIB | dd | no | dd | - | (Bartholomew and Hudson 1960) |
| *Xerospermophilus tereticaudus* | subtropical | HIB | dd | yes | dd | yes | (Bickler 1984; Pengelley and Kelly 1966) |
| *Zapus hudsonius* | temperate | HIB | dd | yes | yes | yes | (Brem et al. 2021; French and Forand 2000) |
| *Zapus princeps* | temperate | HIB | dd | no | yes | yes | (Cranford 1978; French 1985; Luoma 1970) |
| **Soricomorpha** |  |  |  |  |  |  |  |
| *Crocidura flavescens* | subtropical | DT | - | dd | dd | - | (Baxter 1996) |
| *Crocidura leucodon* | temperate | dd | dd | dd | dd | dd | (Genoud 1988; Nagel 1985) |
| *Crocidura russula* | temperate | DT | - | no | no | - | (Nagel 1977; Nagel 1985) |
| *Crocidura suaveolens* | temperate | dd | dd | dd | dd | dd | (Nagel 1985) |
| *Notiosorex crawfordi* | subtropical | DT | - | dd | dd | - | (Lindstedt 1980) |
| *Sorex sinuosus* | subtropical | DT | - | dd | dd | - | (Newman and Rudd 1978) |
| *Suncus etruscus* | subtropical | DT | - | dd | dd | - | (Frey 1979) |
| *Suncus murinus* | tropical | DT | - | yes | dd | - | (Horii et al. 2022; Ishii et al. 2002) |
| **Xenarthra** |  |  |  |  |  |  |  |
| *Zaedyus pichiy* | temperate | HIB | yes | no | no | yes | (Superina and Boily 2007) |

**References**

Alston JM, Dillon ME, Keinath DA, Abernethy IM, Goheen JR. 2022. Daily torpor reduces the energetic consequences of microhabitat selection for a widespread bat. Ecology 103(6):e3677.

Anderson MD. 2004. Aardwolf adaptations: a review. Transactions of the Royal Society of South Africa 59:99-104.

Andriambeloson JB, Greene LK, Blanco MB. 2020. Prolonged torpor in goodman's mouse lemur (*Microcebus lehilahytsara*) from the high-altitude forest of Tsinjoarivo, Central-Eastern Madagascar. Folia Primatologica 91(6):697-710.

Arlettaz R, Ruchet C, Aeschimann J, Brun E, Genoud M, Vogel P. 2000. Physiological traits affecting the distribution and wintering strategy of the bat *Tadarida teniotis*. Ecology 81(4):1004-1014.

Arnold J. 1976. Growth and bioenergetics of the Chuditch, *Dasyurus geoffroii*. University of Western Australia.

Arnold W, Ruf T, Frey-Roos F, Bruns U. 2011. Diet-independent remodeling of cellular membranes precedes seasonally changing body temperature in a hibernator. PLOS ONE 6(4):e18641.

Atherton RG, Haffenden AT. 1982. Observations on the reproduction and growth of the Long-tailed Pygmy Possum, Cercartetus caudatus (Marsupialia: Burramyidae), in captivity. Australian Mammalogy 5(4):253-259.

Atsalis S. 1999. Seasonal fluctuations in body fat and activity levels in a rain-forest species of mouse lemur, *Microcebus rufus*. International Journal of Primatology 20(6):883-910.

Audet D, Thomas DW. 1997. Facultative hypothermia as a thermoregulatory strategy in the phyllostomid bats, *Carollia perspicillata* and *Sturnira lilium*. Journal of Comparative Physiology B 167(2):146-152.

Bakko EB, Nahorniak J. 1986. Torpor Patterns in Captive White-Tailed Prairie Dogs (Cynomys leucurus). Journal of Mammalogy 67(3):576-578.

Barak O, Geiser F, Kronfeld-Schor N. 2018. Flood-induced multiday torpor in golden spiny mice (*Acomys russatus*). Australian Journal of Zoology 66(6):401-405.

Barker JM, Cooper CE, Withers PC, Cruz-Neto AP. 2012. Thermoregulation by an Australian murine rodent, the ash-grey mouse (*Pseudomys albocinereus*). Comparative Biochemistry and Physiology Part A: Molecular & Integrative Physiology 163(3–4):336-342.

Barratt AE, Gonsalves L, Turbill C. 2022. Winter torpor and activity patterns of a fishing bat (*Myotis macropus*) in a mild climate. Journal of Mammalogy 104(1):76-85.

Bartels W, Law BS, Geiser F. 1998. Daily torpor and energetics in a tropical mammal, the nothern blossom-bat *Macroglossus minimus* (Megachiroptera). Journal of Comparative Physiology B 168:233-239.

Bartholomew GA, Cade TJ. 1957. Temperature regulation, hibernation, and aestivation in the little pocket mouse, *Perognathus longimembris*. Journal of Mammalogy 38(1):60-72.

Bartholomew GA, Dawson WR, Lasiewski RC. 1970. Thermoregulation and heterothermy in some of the smaller flying foxes (Megachiroptera) of New Guinea. Zeitschrift für vergleichende Physiologie 70(2):196-209.

Bartholomew GA, Hudson JW. 1960. Aestivation in the Mohave ground squirrel *Citellus mohavensis*. Bulletin of the Museum of Comparative Zoology 124:193-208.

Bartholomew GA, MacMillen RE. 1961. Oxygen consumption, estivation, and hibernation in the kangaroo mouse, *Microdipodops pallidus*. Physiological Zoology 34(3):177-183.

Batavia M, Nguyen G, Harman K, Zucker I. 2013. Hibernation patterns of Turkish hamsters: influence of sex and ambient temperature. J Comp Physiol B 183(2):269-77.

Baxter R. 1996. Evidence for spontaneous torpor in *Crocidura flavescens*. Acta Theriologica 41:327-330.

Bennett N, Jarvis J, Cotteril F. 1993. Poikilothermic traits and thermoregulation in the Afrotropical social subterranean Mashona mole-rat (*Cryptomys hottentotus darlingi*) (Rodentia: Bathyergidae). Journal of Zoology 231:179-186.

Bethge J, Razafimampiandra JC, Wulff A, Dausmann KH. 2021. Sportive lemurs elevate their metabolic rate during challenging seasons and do not enter regular heterothermy. Conserv Physiol 9(1):coab075.

Bickler P. 1984. CO2 balance of a heterothermic rodent: comparison of sleep, torpor, and awake states. American Journal of Physiology-Regulatory, Integrative and Comparative Physiology 246(1):R49-R55.

Blanco M, Rahalinarivo V. 2010. First direct evidence of hibernation in an eastern dwarf lemur species (*Cheirogaleus crossleyi*) from the high-altitude forest of Tsinjoarivo, central-eastern Madagascar. Naturwissenschaften 97:945-950.

Blanco MB, Dausmann KH, Ranaivoarisoa JF, Yoder AD. 2013. Underground hibernation in a primate. Scientific Reports 3:1768.

Bondarenco A, Körtner G, Geiser F. 2014. Hot bats: extreme thermal tolerance in a desert heat wave. Naturwissenschaften 101(8):679-685.

Bondarenco A, Körtner G, Geiser F. 2016. How to keep cool in a hot desert: Torpor in two species of free-ranging bats in summer. Temperature 3(3):476-483.

Boyles JG, Smit B, McKechnie AE. 2012a. Variation in body temperature is related to ambient temperature but not experimental manipulation of insulation in two small endotherms with different thermoregulatory patterns. Journal of Zoology 287(3):224-232.

Boyles JG, Smit B, Sole CL, McKechnie AE. 2012b. Body temperature patterns in two syntopic elephant shrew species during winter. Comparative Biochemistry and Physiology Part A: Molecular & Integrative Physiology 161(1):89-94.

Bozinovic F, Marquet PA. 1991. Energetics and torpor in the atacama desert-dwelling rodent *Phyllotis darwini rupestris*. Journal of Mammalogy 72(4):734-738.

Bozinovic F, Rosenmann M. 1988. Daily torpor in *Calomys musculinus*, a South American rodent. Journal of Mammalogy 69(1):150-152.

Bozinovic F, RuÍz G, CortÉs A, Rosenmann M. 2005. Energetics, thermoregulation and torpor in the Chilean mouse-opossum Thylamys elegans (Didelphidae). Revista chilena de historia natural 78:199-206.

Brem EA, McNulty AD, Israelsen WJ. 2021. Breeding and hibernation of captive meadow jumping mice (*Zapus hudsonius*). PLOS ONE 16(5):e0240706.

Brown JH, Bartholomew GA. 1969. Periodicity and energetics of torpor in the kangaroo mouse, *Microdipodops pallidus*. Ecology 50(4):705-709.

Buffenstein R, Jarvis JUM. 1985. Thermoregulation and metabolism in the smallest African gerbil, *Gerbillus pusillus*. Journal of Zoology 205(1):107-121.

Burbidge A, McKenzie N, Fuller P. 2008. Long-tailed dunnart, *Sminthopsis longicaudata*. . In: van Dyke S, Strahan R, editors. The mammals of Australia. Sydney: Reed New Holland. p. 148-150.

Busse S, Lutter D, Heldmaier G, Jastroch M, Meyer CW. 2014. Torpor at high ambient temperature in a neotropical didelphid, the grey short-tailed opossum (Monodelphis domestica). Naturwissenschaften 101(11):1003-1006.

Cade T. 1964. The evolution of torpidity in rodents. Annales Academiae Scientiarum Fennicae A IV Biologica 71:77-111.

Caviedes-Vidal E, Codelia EC, Roig V, Doña R. 1990. Facultative torpor in the south american rodent *Calomys venustus* (Rodentia: Cricetidae). Journal of Mammalogy 71(1):72-75.

Channing A. 1984. Ecology of the namtap *Graphiurus ocularis* (Rodentia:Gliridae) in the Cedarberg, South Africa. African Zoology 19(3):144-149.

Chi Q-S, Wan X-R, Geiser F, Wang D-H. 2016. Fasting-induced daily torpor in desert hamsters (*Phodopus roborovskii*). Comparative Biochemistry and Physiology Part A: Molecular & Integrative Physiology 199:71-77.

Christian N, Geiser F. 2007. To use or not to use torpor? Activity and body temperature as predictors. Naturwissenschaften 94(6):483-487.

Coburn DK, Geiser F. 1998. Seasonal changes in energetics and torpor patterns in the subtropical blossom-bat *Syconycteris australis* (Megachiroptera). Oecologia 113(4):467-473.

Çolak E, Yi̇ği N. 1998. Ecology and biology of Allactaga elater, *Allactaga euphratica* and *Allactaga williamsi* (Rodentia: Dipodidae) in Turkey. Turkish Journal of Zoology 22:105-118.

Collins B, Wooler R, Richardson K. 1987. Torpor in the honey possum, *Tarsipes rostratus* (Marsupialia: Tarsipedidae). Australian Mammalogy 11:51-57.

Cooper CE, Withers PC. 2004. Patterns of body temperature variation and torpor in the numbat, *Myrmecobius fasciatus* (Marsupialia: Myrmecobiidae). Journal of Thermal Biology 29(6):277-284.

Cooper CE, Withers PC. 2010. Comparative physiology of Australian quolls (Dasyurus; Marsupialia). Journal of Comparative Physiology B 180(6):857-68.

Cooper CE, Withers PC, Cruz-Neto AP. 2009. Metabolic, ventilatory, and hygric physiology of the gracile mouse opossum (*Gracilinanus agilis*). Physiological and Biochemical Zoology 82(2):153-62.

Cooper CE, Withers PC, Cruz-Neto AP. 2010. Metabolic, ventilatory, and hygric physiology of a South American marsupial, the long-furred woolly mouse opossum. Journal of Mammalogy 91(1):1-10.

Cory Toussaint D, McKechnie AE. 2012. Interspecific variation in thermoregulation among three sympatric bats inhabiting a hot, semi-arid environment. Journal of Comparative Physiology B 182(8):1129-1140.

Cory Toussaint D, McKechnie AE, van der Merwe M. 2010. Heterothermy in free-ranging male Egyptian Free-tailed bats (Tadarida aegyptiaca) in a subtropical climate. Mammalian Biology - Zeitschrift für Säugetierkunde 75(5):466-470.

Cranford JA. 1978. Hibernation in the western jumping mouse (*Zapus princeps*). Journal of Mammalogy 59(3):496-509.

Cranford JA. 1986. Basin ground squirrel (*Spermophilus armatus*). In Living in the Cold: Physiological and Biochemical Adaptations: Proceedings of the Seventh International Symposium on Natural Mammalian Hibernation. Elsevier Publishing Company. p. 411.

Cryan PM, Wolf BO. 2003. Sex differences in the thermoregulation and evaporative water loss of a heterothermic bat, *Lasiurus cinereus*, during its spring migration. The Journal of Experimental Biology 206:3381-3390.

Czenze ZJ, Brigham RM, Hickey AJR, Parsons S. 2017. Winter climate affects torpor patterns and roost choice in New Zealand lesser short-tailed bats. Journal of Zoology 303(3):236-243.

Daan S. 1973. Periodicity of heterothermy in the garden dormouse, *Eliomys quercinus* (L.). Netherlands Journal of Zoology 23(3):237-265.

Daniel S, Korine C, Pinshow B. 2010. The use of torpor in reproductive female Hemprich’s long eared bats (*Otonycteris hemprichii*). Physiological and Biochemical Zoology: Ecological and Evolutionary Approaches 83(1):142-148.

Dausmann KH. 2008. Hypometabolism in primates: torpor and hibernation. In: Lovegrove BG, McKechnie AE, editors. Hypometabolism in Animals: Hibernation, Torpor and Cryobiology. Pietermaritzburg, South Africa: Interpak. p. 327-336.

Dausmann KH. 2014. Flexible patterns in energy savings: heterothermy in primates. Journal of Zoology 292(2):101-111.

Dausmann KH, Glos J, Ganzhorn JU, Heldmaier G. 2004. Physiology: hibernation in a tropical primate. Nature 429(6994):825-826.

Dausmann KH, Körtner G, Aharon-Rotman Y, Currie SE, Geiser F. 2023. Flexible employment of torpor in squirrel gliders (*Petaurus norfolcensis*): An adaptation to unpredictable climate? Physiological and Biochemical Zoology 96(1):62-74.

Dausmann KH, Levesque DL, Wein J, Nowack J. 2020. Ambient temperature cycles affect daily torpor and hibernation patterns in Malagasy tenrecs. Front Physiol 11:522.

Davis DE, Swade RH. 1983. Circannual rhythm of torpor and molt in the ground squirrel, *Spermophilus beecheyi*. Comparative Biochemistry and Physiology A 76(1):183-187.

Dawson TJ, Wolfers JM. 1978. Metabolism, thermoregulation and torpor in shrew sized marsupials of the genus planigale. Comparative Biochemistry and Physiology Part A: Physiology 59(3):305-309.

Dunbar MB, Tomasi TE. 2006. Arousal patterns, metabolic rate, and an energy budget of eastern red bats (*Lasiurus borealis*) in winter. Journal of Mammalogy 87(6):1096-1102.

Dzal YA, Brigham RM. 2013. The tradeoff between torpor use and reproduction in little brown bats (*Myotis lucifugus*). Journal of Comparative Physiology B 183(2):279-288.

Eisentraut M. 1956. Der Winterschlaf mit seinen ökologischen und physiologischen Begleiterscheinungen. Jena: Fischer.

El Ouezzani S, Janati IA, Magoul R, Pévet P, Saboureau M. 2011. Overwinter body temperature patterns in captive jerboas (*Jaculus orientalis*): influence of sex and group. Journal of Comparative Physiology B 181(2):299-309.

Eto T, Sakamoto SH, Okubo Y, Koshimoto C, Kashimura A, Morita T. 2014. Huddling facilitates expression of daily torpor in the large Japanese field mouse *Apodemus speciosus*. Physiology & Behavior 133(0):22-29.

Fielden L, Waggoner J, Perrin M, Hickmann G. 1990. Thermoregulation in the Namib Desert golden mole, *Eremitalpa granti namibensis* (Chrysochloridae). Journal of Arid Environments 18:221–37.

Fleming MR. 1985. The thermal physiology of the feathertail glider, *Acrobates pygmaeus* (Marsupialia:Burramyidae). Australian Journal of Zoology 33(5):667-681.

Fowler PA, Racey PA. 1988. Overwintering strategies of the badger, *Meles meles*, at 57 °N. Journal of Zoology 214(4):635-651.

Fowler PA, Racey PA. 1990. Daily and seasonal cycles of body temperature and aspects of heterothermy in the hedgehog *Erinaceus europaeus*. Journal of Comparative Physiology B 160(3):299-307.

Frank CL, Karpovich S, Barnes BM. 2008. Dietary fatty acid composition and the hibernation patterns in free-ranging arctic ground squirrels. Physiological and Biochemical Zoology: Ecological and Evolutionary Approaches 81(4):486-495.

French A. 1985. Allometries of the durations of torpid and euthermic intervals during mammalian hibernation: A test of the theory of metabolic control of the timing of changes in body temperature. Journal of Comparative Physiology B 156(1):13-19.

French AR. 1989. Seasonal Variation in Use of Torpor by Pallid Kangaroo Mice, Microdipodops pallidus. Journal of Mammalogy 70(4):839-842.

French AR, Forand S. Role of soil temperature in timing of emergence from hibernation in the jumping mouse, *Zapus hudsonius*. In: Heldmaier G, Klingenspor M, editors. Life in the Cold; 2000// 2000; Berlin, Heidelberg: Springer Berlin Heidelberg. p. 111-118.

Frey H. 1979. La température corporelle de Suncus etruscus (Soricidae, Insectivora) au cours de l'activité, du repos normothermique et de la torpeur. Revue Suisse de Zoologie 86:653-662.

Geiser F. 1986. Thermoregulation and torpor in the Kultarr,Antechinomys laniger (Marsupialia: Dasyuridae). Journal of Comparative Physiology B 156(5):751-757.

Geiser F. 1987. Hibernation and daily torpor in two pygmy-possums (*Cercartetus spp*., Marsupialia). Physiological Zoology 60(1):93-102.

Geiser F. 1988. Daily torpor and thermoregulation in antechinus (Marsupialia): influence of body mass, season, development, reproduction, and sex. Oecologia 77(3):395-399.

Geiser F. 1991. The effect of unsaturated and saturated dietary lipids on the pattern of daily torpor and the fatty acid composition of tissues and membranes of the deer mouse *Peromyscus maniculatus*. Journal of Comparative Physiology B 161(6):590-597.

Geiser F, Augee ML, McCarron HCK, Raison JK. 1984 Correlates of torpor in the insectivorous dasyurid marsupial *Sminthopsis murina*. Australian Mammalogy 7:185-191.

Geiser F, Baudinette R. 1988. Daily torpor and thermoregulation in the small dasyurid marsupials *Planigale gilesi* and *Ningaui yvonneae*. Australian Journal of Zoology 36(4):473-481.

Geiser F, Baudinette RV. 1987. Seasonality of torpor and thermoregulation in three dasyurid marsupials. Journal of Comparative Physiology B 157(3):335-344.

Geiser F, Brigham RM. 2000. Torpor, thermal biology, and energetics in Australian long-eared bats (Nyctophilus). Journal of Comparative Physiology B 170(2):153-162.

Geiser F, Broome LS. 1993. The effect of temperature on the pattern of torpor in a marsupial hibernator. Journal of Comparative Physiology B 163(2):133-137.

Geiser F, Cooper CE. 2023. Daily Torpor, Hibernation, and Heterothermy in Marsupials. In: Cáceres NC, Dickman CR, editors. American and Australasian Marsupials: An Evolutionary, Biogeographical, and Ecological Approach. Cham: Springer International Publishing. p. 1-28.

Geiser F, Hiebert S, Kenagy GJ. 1990. Torpor bout duration during the hibernation season of two sciurid rodents: Interrelations with temperature and metabolism. Physiological Zoology 63(3):489-503.

Geiser F, Körtner G. 2010. Hibernation and daily torpor in Australian mammals. Australian Zoologist 35(2):204-215.

Geiser F, Martin G. 2013. Torpor in the Patagonian opossum (*Lestodelphys halli*): implications for the evolution of daily torpor and hibernation. Naturwissenschaften 100(10):975-981.

Geiser F, Masters P. 1994. Torpor in relation to reproduction in the Mulgara, *Dasycercus cristicauda* (Dasyuridae: Marsupilia). Journal of Thermal Biology 19(1):33-40.

Geiser F, Mzilikazi N. 2011. Does torpor of elephant shrews differ from that of other heterothermic mammals? Journal of Mammalogy 92(2):452-459.

Geiser F, Pavey CR. 2007. Basking and torpor in a rock-dwelling desert marsupial: survival strategies in a resource-poor environment. Journal of Comparative Physiology B 177(8):885-892.

Geiser F, Stawski C. 2011. Hibernation and torpor in tropical and subtropical bats in relation to energetics, extinctions, and the evolution of endothermy. Integrative and Comparative Biology 51(3):337-348.

Genoud M. 1988. Energetic strategies of shrews: ecological constraints and evolutionary implications. Mammal Review 18(4):173-193.

Genoud M, Bonaccorso FJ, Anends A. 1990. Rate of metabolism and temperature regulation in two small tropical insectivorous bats (*Peropteryx macrotis* and *Natalus tumidirostris*). Comparative Biochemistry and Physiology Part A: Physiology 97(2):229-234.

Genoud M, Christe P. 2011. Thermal energetics and torpor in the common pipistrelle bat, *Pipistrellus pipistrellus* (Vespertilionidae: Mammalia). Comparative Biochemistry and Physiology Part A: Molecular & Integrative Physiology 160(2):252-259.

Giroud S, Blanc S, Aujard F, Bertrand F, Gilbert C, Perret M. 2008. Chronic food shortage and seasonal modulations of daily torpor and locomotor activity in the grey mouse lemur (*Microcebus murinus*). American Journal of Physiology-Regulatory, Integrative and Comparative Physiology 294(6):R1958-R1967.

Giroud S, Frare C, Strijkstra A, Boerema A, Arnold W, Ruf T. 2013. Membrane phospholipid fatty acid composition regulates cardiac serca activity in a hibernator, the syrian hamster (*Mesocricetus auratus*). PLOS ONE 8(5):e63111.

Giroud S, Zahn S, Criscuolo F, Chery I, Blanc S, Turbill C, Ruf T. 2014. Late-born intermittently fasted juvenile garden dormice use torpor to grow and fatten prior to hibernation: consequences for ageing processes. Proceedings of the Royal Society of London B: Biological Sciences 281(1797).

Gottfried I. 2009. Use of underground hibernacula by the barbastelle (*Barbastella barbastellus*) outside the hibernation season. Acta Chiropterologica 11(2):363-373, 11.

Gotts D. 1976. Energetics in *Antechinus swainsonii*. Monash University, Melbourne.

Grigg G, Beard L. 2000. Hibernation by echidnas in mild climates: hints about the evolution of endothermy? In: Heldmaier G, Klingenspor M, editors. Life in the Cold. Springer Berlin Heidelberg. p. 5-19.

Gummer DL. 2005. Geographic variation in torpor patterns: The northernmost prairie dogs and kangaroo rats. [Saskatoon]: University of Saskatchewan.

Gyhrs C, Macedo T, Bastos B, Salgado-Irazabal X, Hammadi M, Bouarakia O, Boratyński Z. 2022. High level of daily heterothermy in desert gerbils. Journal of Tropical Ecology 38(6):451-453.

Hall LS. 1982. The effect of cave microclimate on winter roosting behaviour in the bat, *Miniopterus schreibersii blepotis*. Australian Journal of Ecology 7(2):129-136.

Hallam SL, Mzilikazi N. 2011. Heterothermy in the southern African hedgehog, *Atelerix frontalis*. Journal of Comparative Physiology B 181(3):437-445.

Harlow HJ. 1981. Torpor and other physiological adaptations of the badger (*Taxidea taxus)* to cold environments. Physiological Zoology 54(3):267-275.

Harlow HJ, Jr. GEM. 1986. A comparison of hibernation in the black-tailed prairie dog, white-tailed prairie dog, and Wyoming ground squirrel. Canadian Journal of Zoology 64(3):793-796.

Harmata W. 1987. The frequency of winter sleep interruptions in two species of bats hibernating in limestone tunnels. Acta Theriologica 32:331-332.

Haugg E, Herwig A, Diedrich V. 2021. Body temperature and activity adaptation of short photoperiod-exposed djungarian hamsters (*Phodopus sungorus*): Timing, traits, and torpor. Front Physiol 12:626779.

Healy JE, Burdett KA, Buck CL, Florant GL. 2012. Sex differences in torpor patterns during natural hibernation in golden-mantled ground squirrels (Callospermophilus lateralis). Journal of Mammalogy 93(3):751-758.

Herreid C, 2nd, Schmidt-Nielsen K. 1966. Oxygen consumption, temperature, and water loss in bats from different environments. American Journal of Physiology-Legacy Content 211(5):1108-1112.

Hill VL, Florant GL. 2000. The effect of a linseed oil diet on hibernation in yellow-bellied marmots (*Marmota flaviventris*). Physiology & Behavior 68(4):431-437.

Hissa R, Siekkinen J, Hohtola E, Saarela S, Hakala A, Pudas J. 1994. Seasonal patterns in the physiology of the European brown bear (*Ursus arctos arcto*s) in Finland. Comparative Biochemistry and Physiology Part A: Physiology 109(3):781-791.

Hoelzl F, Bieber C, Cornils JS, Gerritsmann H, Stalder GL, Walzer C, Ruf T. 2015. How to spend the summer? Free-living dormice (*Glis glis)* can hibernate for 11 months in non-reproductive years. Journal of Comparative Physiology B 185(8):931-939.

Hope PR, Jones G. 2012. Warming up for dinner: torpor and arousal in hibernating Natterer's bats (*Myotis nattereri*) studied by radio telemetry. Journal of Comparative Physiology B 182(4):569-78.

Horii Y, Okadera K, Miyawaki S, Shiina T, Shimizu Y. 2022. *Suncus murinus* as a novel model animal that is suitable for elucidating the mechanism of daily torpor. Biomed Res 43(2):53-57.

Hosken DJ, Withers PC. 1997. Temperature regulation and metabolism of an Australian bat, *Chalinolobus gouldii* (Chiroptera: Vespertilionidae) when euthermic and torpid. Journal of Comparative Physiology B 167(1):71-80.

Hudson JW. 1965. Temperature regulation and torpidity in the pygmy mouse, *Baiomys taylori*. Physiological Zoology 38(3):243-254.

Hudson JW, Deavers DR. 1973. Thermoregulation at high ambient temperatures of six species of ground squirrels (*Spermophilus spp*.) from different habitats. Physiological Zoology 46(2):95-109.

Hwang YT, Larivière S, Messier F. 2007. Energetic consequences and ecological significance of heterothermy and social thermoregulation in striped skunks (*Mephitis mephitis*). Physiological and Biochemical Zoology 80(1):138-145.

Ishii K, Uchino M, Kuwahara M, Tsubone H, Ebukuro S. 2002. Diurnal fluctuations of heart rate, body temperature and locomotor activity in the house musk shrew (*Suncus murinus*). Exp Anim 51(1):57-62.

Jackson CR, Setsaas TH, Robertson MP, Scantlebury M, Bennett NC. 2009. Insights into torpor and behavioural thermoregulation of the endangered Juliana's golden mole. Journal of Zoology 278(4):299-307.

Jacobs DS, Kelly EJ, Mason M, Stoffberg S. 2007. Thermoregulation in two free-ranging subtropical insectivorous bat species: Scotophilus species (Vespertilionidae). Canadian Journal of Zoology 85(8):883-890.

Johnson JS. 2012. Foraging and roosting behaviors of rafifinesque's big-eared bat (*Corynorhinus rafifinesquii*) at the northern edge of the species range. [Theses and Dissertations--Animal and Food Sciences 5]: University of Kentucky.

Jones CJ, Geiser F. 1992. Prolonged and daily torpor in the feathertail glider, *Acrobates pygmaeus* (Marsupialia: Acrobatidae). Journal of Zoology 227(1):101-108.

Kart Gür M, Bulut Ş, Gür H, Refinetti R. 2014. Body temperature patterns and use of torpor in an alpine glirid species, woolly dormouse. Acta Theriologica 59(2):299-309.

Kart Gür M, Refinetti R, Gür H. 2009. Daily rhythmicity and hibernation in the Anatolian ground squirrel under natural and laboratory conditions. J Comp Physiol B 179(2):155-64.

Kawamichi M. 1993. Factors affecting hibernation commencement and spring emergence in Siberian chipmunks (*Eutamias sibiricus*). In: Carey C, Florant G, Wunder B, Horwitz B, editors. Life in the cold: ecological, physiological and molecular mechanisms. Boulder, Colorado: Westview. p. 81-89.

Keicher L, Shipley JR, Komar E, Ruczyński I, Schaeffer PJ, Dechmann DKN. 2022. Flexible energy-saving strategies in female temperate-zone bats. Journal of Comparative Physiology B 192(6):805-814.

Kelm D, Helversen O. 2007. How to budget metabolic energy: torpor in a small Neotropical mammal. Journal of Comparative Physiology B 177(6):667-677.

Kelt D, Martinez D. 1989. otes on distribution and ecology of two marsupials endemic to the valdivian forests of southern South America. Journal of Mammology 70:220-224.

Khrushchova AM, Vasilieva NY, Shekarova ON, Rogovin KA, Petrovski DV. 2018. Torpor in dwarf hamsters, *Phodopus campbelli* and *Phodopus roborovski*i: a comparative study. 6th Internatioal Conference of Rodent Biology ad Management and 16th Rodens et Spatium Potsdam, Germany.

Kisser B, Goodwin HT. 2012. Hibernation and overwinter body temperatures in free-ranging thirteen-lined ground squirrels, ictidomys tridecemlineatus. The American Midland Naturalist 167(2):396-409, 14.

Kobbe S, Ganzhorn J, Dausmann KH. 2011. Extreme individual flexibility of heterothermy in free-ranging Malagasy mouse lemurs (*Microcebus griseorufus*). Journal of Comparative Physiology B: Biochemical, Systemic, and Environmental Physiology 181(1):165-173.

Körtner G, Geiser F. 2009. The key to winter survival: daily torpor in a small arid-zone marsupial. Naturwissenschaften 96(4):525-530.

Körtner G, Geiser F. 2011. Activity and torpor in two sympatric Australian desert marsupials. Journal of Zoology 283(4):249-256.

Körtner G, Pavey CR, Geiser F. 2008. Thermal biology, torpor, and activity in free‐living mulgaras in arid zone Australia during the winter reproductive season. Physiological and Biochemical Zoology 81(4):442-451.

Körtner G, Riek A, Pavey CR, Geiser F. 2016. Activity patterns and torpor in two free-ranging carnivorous marsupials in arid Australia in relation to precipitation, reproduction, and ground cover. Journal of Mammalogy 97(6):1555-1564.

Körtner G, Rojas AD, Geiser F. 2010. Thermal biology, torpor use and activity patterns of a small diurnal marsupial from a tropical desert: sexual differences. Journal of Comparative Physiology B 180(6):869-876.

Kulzer E. 1965. Temperaturregulation bei Fledermäusen (Chiroptera) aus verschiedenen Klimazonen. Zeitschrift für vergleichende Physiologie 50:1-34.

Kulzer E, Nelson J, McKean J, Möhres P. 1970. Untersuchungen über die Temperaturregulation australischer Fledermäuse. Zeitschrift für vergleichende Physiologie 69:426–51.

Kulzer E, Storf R. 1980. Sleep-lethargy in the African long-tongued fruit bat *Megaloglossus woermanni pagenstecher*, 1885. Zeitschrift fur Saugetierkunde-International Journal of Mammalian Biology 45(1):23-29.

Lahann P. 2007. Biology of *Cheirogaleus major* in a littoral rain forest in Southeast Madagascar. International Journal of Primatology 28(4):895-905.

Lahann P, Dausmann K. 2011. Live fast, die young: flexibility of life history traits in the fat-tailed dwarf lemur (*Cheirogaleus medius*). Behavioral Ecology and Sociobiology 65:381–390.

Lee TN, Barnes BM, Buck CL. 2009. Body temperature patterns during hibernation in a free-living Alaska marmot (*Marmota broweri*). Ethology Ecology & Evolution 21(3-4):403-413.

Lehmer EM, Biggins DE. 2005. Variation in Torpor Patterns of Free-Ranging Black-Tailed and Utah Prairie Dogs Across Gradients of Elevation. Journal of Mammalogy 86(1):15-21.

Lehmer EM, Van Horne B, Kulbartz B, Florant GL. 2001. Facultative Torpor in Free-Ranging Black-Tailed Prairie Dogs (Cynomys Ludovicianus). Journal of Mammalogy 82(2):551-557.

Levesque DL, Lovegrove BG. 2014. Increased homeothermy during reproduction in a basal placental mammal. Journal of Experimental Biology 217(9):1535-1542.

Levesque DL, Tattersall GJ. 2010. Seasonal torpor and normothermic energy metabolism in the Eastern chipmunk (*Tamias striatus*). Journal of Comparative Physiology B 180(2):279-292.

Levin E, Ar A, Yom-Tov Y, Kronfeld-Schor N. 2012. Summer torpor and sexual segregation in the subtropical bat *Rhinopoma microphyllum*. In: Ruf T, Bieber C, Arnold W, Millesi E, editors. Living in a Seasonal World: Thermoregulatory and Metabolic Adaptations. Berlin, Heidelberg: Springer Berlin Heidelberg. p. 167-174.

Levin E, Plotnik B, Amichai E, Braulke LJ, Landau S, Yom-Tov Y, Kronfeld-Schor N. 2015. Subtropical mouse-tailed bats use geothermally heated caves for winter hibernation. Proceedings of the Royal Society B: Biological Sciences 282(1804):20142781.

Levy O, Dayan T, Kronfeld-Schor N. 2011. Interspecific competition and torpor in golden spiny mice: two sides of the energy-acquisition coin. Integrative and Comparative Biology 51(3):441-448.

Lindstedt SL. 1980. Regulated hypothermia in the desert shrew. Journal of Comparative Physiology 137(2):173-176.

Liu J-N, Karasov WH. 2011. Hibernation in warm hibernacula by free-ranging Formosan leaf-nosed bats, *Hipposideros terasensis*, in subtropical Taiwan. Journal of Comparative Physiology B 181(1):125-135.

Liu J-N, Karasov WH. 2012. Metabolism during winter in a subtropical hibernating bat, the Formosan leaf-nosed bat (*Hipposideros terasensis*). Journal of Mammalogy 93(1):220-228.

Lovegrove BG, Canale CI, Levesque DL, Fluch G, Reháková-Petrů M, Ruf T. 2014a. Are tropical small mammals physiologically vulnerable to Arrhenius effects and climate change? . Physiological and Biochemical Zoology 87(1):30-45.

Lovegrove BG, Génin F. 2008. Torpor and hibernation in a basal placental mammal, the lesser hedgehog tenrec *Echinops telfairi*. Journal of Comparative Physiology, B: Biochemical, Systematic, and Environmental Physiology 168:303-312.

Lovegrove BG, Lawes MJ, Roxburgh L. 1999. Confirmation of pleisiomorphic daily torpor in mammals: the round-eared elephant shrew *Macroscelides proboscideus* (Macroscelidea). Journal of Comparative Physiology B: Biochemical, Systemic, and Environmental Physiology 169(7):453-460.

Lovegrove BG, Lobban KD, Levesque DL. 2014b. Mammal survival at the Cretaceous–Palaeogene boundary: metabolic homeostasis in prolonged tropical hibernation in tenrecs. Proceedings of the Royal Society B: Biological Sciences 281(1796): 20141304.

Lovegrove BG, Raman J. 1998. Torpor patterns in the pouched mouse (*Saccostomus campestris*; Rodentia): a model animal for unpredictable environments. J Comp Physiol B 168(4):303-12.

Lovegrove BG, Raman J, Perrin MR. 2001. Heterothermy in elephant shrews, *Elephantulus spp.* (Macroscelidea): daily torpor or hibernation? J Comp Physiol B 171(1):1-10.

Luoma SN. 1970. A study of hibernation in the western jumping mouse, *Zapus princeps*. Montana State University.

Lyman C, O'Brien R, Greene G, Papafrangos E. 1981. Hibernation and longevity in the Turkish hamster *Mesocricetus brandti*. Science 212(4495):668-670.

MacMillen R. 1965. Aestivation in the cactus mouse, *Peromyscus eremicus*. Comparative Biochemistry and Physiology - Part A: Molecular & Integrative Physiology 16(2):227-248.

MacMillen RE. 1983. Adaptive physiology of heteromyid rodents. Great Basin Naturalist Memoirs(7):65-76.

Marom S, Korine C, Wojciechowski MS, Tracy CR, Pinshow B. 2006. Energy metabolism and evaporative water loss in the European free‐tailed bat and Hemprich’s long‐eared bat (Microchiroptera): Species sympatric in the Negev desert. Physiological and Biochemical Zoology: Ecological and Evolutionary Approaches 79(5):944-956.

Masaki M, Koshimoto C, Tsuchiya K, Nishiwaki A, Morita T. 2005. Body temperature profiles of the Korean field mouse Apodemus peninsulae during winter aggregation. Mammal Study 30(1):33-40.

McNab BK. 1978a. The comparative energetics of neotropical marsupials. Journal of Comparative Physiology 125(2):115-128.

McNab BK. 1978b. The evolution of endothermy in the phylogeny of mammals. The American Naturalist 112(983):1-21.

McNab BK, Bonaccorso FJ. 2001. The metabolism of New Guinean pteropodid bats. Journal of Comparative Physiology B 171(3):201-214.

Morhardt EJ. 1970. Body temperatures of white-footed mice (*Peromyscus sp*.) during daily torpor. Comparative Biochemistry and Physiology 33(2):423-439.

Morrison PR, McNab BK. 1962. Daily torpor in a brazilian murine opossum (marmosa). Comparative Biochemistry and Physiology 6:57-68.

Morton SR, Lee AK. 1978. Thermoregulation and metabolism in *Planigale maculata* (Marsupialia: Dasyuridae). Journal of Thermal Biology 3(3):117-120.

Mouhoub-Sayah C, Robin J-P, Malan A, Pevet P, Saboureau M. 2008. Patterns of body temperature change in the Algerian hedgehog (*Atelerix algirus*). In: Lovegrove BG, McKechnie AE, editors. Hypometabolism in Animals: Torpor, Hibernation and Cryobiology. 13th International Hibernation Symposium. Pietermaritzburg: University of KwaZulu-Natal. p. 307–316.

Müller EF, Nieschalk U, Meier B. 1985. Thermoregulation in the slender loris (*Loris tardigradus*). Folia Primatologica 44(3-4):216-226.

Muller J. 1996. Torpor in *Sminthopsis douglasi*. La Trobe University, Bundoora, Victoria.

Muñoz-Garcia A, Ben-Hamo M, Pinshow B, Williams JB, Korine C. 2012. The relationship between cutaneous water loss and thermoregulatory state in Kuhl's pipistrelle *Pipistrellus kuhlii*, a Vespertillionid bat. Physiol Biochem Zool 85(5):516-25.

Muul I. 1968. Behavioural and physiological influences on the distribution of the flying squirrel, *Glaucomys volans*. University of Michigan, Museum of Zoology, Miscellaneous Publication 134:1-65.

Mzilikazi N, Lovegrove B. 2002. Reproductive activity influences thermoregulation and torpor in pouched mice, *Saccostomus campestris*. Journal of Comparative Physiology, B: Biochemical, Systematic, and Environmental Physiology 172:7-16.

Mzilikazi N, Lovegrove BG. 2004. Daily torpor in free-ranging rock elephant shrews, *Elephantulus myurus*: a year-long study. Physiological and Biochemical Zoology 77(2):285–296.

Mzilikazi N, Lovegrove BG. 2005. Daily torpor during the active phase in free-ranging rock elephant shrews (*Elephantulus myurus)*. Journal of Zoology 267(01):103-111.

Mzilikazi N, Madikiza Z, Oelkrug R, Baxter R. 2012. Hibernation in free-ranging African woodland dormice, *Graphiurus murinus*. In: Ruf T, Bieber C, Arnold W, Millesi E, editors. Living in a Seasonal World. Springer Berlin Heidelberg. p. 41-50.

Nagel A. 1977. Torpor in the European white-toothed shrews. Experientia 33(11):1455-1456.

Nagel A. 1985. Sauerstoffverbrauch, Temperaturregulation und Herzfrequenz bei europäischen Spitzmäusen (Soricidae). Zeitschrift für Säugetierkunde 50:249-266.

Németh I, Nyitrai V, Németh A, Altbäcker V. 2010. Diuretic treatment affects the length of torpor bouts in hibernating European ground squirrels (*Spermophilus citellus*). J Comp Physiol B 180(3):457-64.

Nespolo RF, Mejías C, Espinoza A, Quintero-Galvis J, Rezende EL, Fontúrbel FE, Bozinovic F. 2021. Heterothermy as the Norm, Homeothermy as the Exception: Variable Torpor Patterns in the South American Marsupial Monito del Monte (Dromiciops gliroides). Frontiers in Physiology 12.

Neumann RL, Cade TJ. 1965. Torpidity in the mexican ground squirrel *Citellus mexicanus parvidens* (mearns). Canadian Journal of Zoology 43:133-40.

Nevo E, Amir E. 1964. Geographic Variation in Reproduction and Hibernation Patterns of the Forest Dormouse. Journal of Mammalogy 45(1):69-87.

Newman BA. 2020. Winter torpor and roosting ecology of tri-colored bats (*Perimyotis subflavus*) in trees and bridges. Clemson University.

Newman J, Rudd R. 1978. Minimum and maximum metabolic rates of *Sorex sinuosus*. Acta Theriologica 23:371-380.

Nowack J, Cooper CE, Geiser F. 2016. Cool echidnas survive the fire. Proceedings of the Royal Society of London B: Biological Sciences 283(1828):20160382.

Nowack J, Geiser F. 2016. Friends with benefits: the role of huddling in mixed groups of torpid and normothermic animals. Journal of Experimental Biology 219(4):590-596.

Nowack J, Mzilikazi N, Dausmann KH. 2013. Torpor as an emergency solution in *Galago moholi*: heterothermy is triggered by different constraints Journal of Comparative Physiology B 183:547-556.

Nowack J, Rojas AD, Körtner G, Geiser F. 2015. Snoozing through the storm: torpor use during a natural disaster. Scientific Reports 5:11243.

Nowack J, Turbill C. 2022. Survivable hypothermia or torpor in a wild-living rat: rare insights broaden our understanding of endothermic physiology. J Comp Physiol B 192(1):183-192.

O'Farrell MJ, Studier EH. 1970. Fall metabolism in relation to ambient temperatures in three species of Myotis. Comparative Biochemistry and Physiology 35(3):697-703.

O'Mara MT, Rikker S, Wikelski M, Ter Maat A, Pollock HS, Dechmann DKN. 2017. Heart rate reveals torpor at high body temperatures in lowland tropical free-tailed bats. Royal Society Open Science 4(12):171359.

Oelkrug R, Meyer C, Heldmaier G, Mzilikazi N. 2012. Seasonal changes in thermogenesis of a free-ranging afrotherian small mammal, the western rock elephant shrew (*Elephantulus rupestris*). Journal of Comparative Physiology B: Biochemical, Systemic, and Environmental Physiology:1-13.

Olson MN, Bowman J, Burness G. 2017. Seasonal energetics and torpor use in North American flying squirrels. Journal of Thermal Biology 70:46-53.

Opazo JC, Nespolo RF, Bozinovic F. 1999. Arousal from torpor in the chilean mouse-opposum (*Thylamys elegans*): does non-shivering thermogenesis play a role? Comparative Biochemistry and Physiology Part A: Comparative Physiology 123:393-397.

Ortmann S, Heldmaier G, Schmid J, Ganzhorn JU. 1997. Spontaneous daily torpor in Malagasy mouse lemurs. Naturwissenschaften 84(1):28-32.

Otsu R, Kimura T. 1993. Effects of food availability and ambient temperature on hibernation in the Japanese dormouse,Glirulus japonicus. Journal of Ethology 11(1):37-42.

Park KJ, Jones G, Ransome RD. 2000. Torpor, arousal and activity of hibernating greater horseshoe bats (*Rhinolophus ferrumequinum*). Functional Ecology 14(5):580-588.

Pengelley ET. 1964. Responses of a new hibernator (*Citellus variegatus*) to controlled environments. Nature 203:892-892.

Pengelley ET, Kelly KH. 1966. A “circannian” rhythm in hibernating species of the genus Citellus with observations on their physiological evolution. Comparative Biochemistry and Physiology 19(3):603-617.

Perrin MR, Richardson EJ. 2004. Factors affecting the induction of torpor and body mass in the fat mouse *Steatomys pratensis*. Journal of Thermal Biology 29(3):133-139.

Perrin MR, Ridgard BW. 1999. Thermoregulation and patterns of torpor in the spectacled dormouse, *Graphiurus ocularis* (A. Smith 1829) (Gliridae). Tropical Zoology 12(2):253-266.

Pohl H. 1961. Temperaturregulation und Tagesperiodik des Stoffwechsels bei Winterschläfern. Zeitschrift für Vergleichende Physiologie 45(2):109-153.

Pretorius M, Markotter W, Kearney T, Seamark E, Broders H, Keith M. 2021. No evidence of pre-hibernation or pre-migratory body mass gain in *Miniopterus natalensis* in north-eastern south africa. Journal of Vertebrate Biology 70(1).

Pretzlaff I, Dausmann K. 2012. Impact of climatic variation on the hibernation physiology of *Muscardinus avellanarius*. In: Ruf T, Bieber C, Arnold W, Millesi E, editors. Living in a Seasonal World. Springer Berlin Heidelberg. p. 85-97.

Pretzlaff I, Rau D, H. Dausmann K. 2014. Energy expenditure increases during the active season in the small, free-living hibernator *Muscardinus avellanarius*. Mammalian Biology 79(3):208-214.

Przybylska-Piech AS, Jefimow M. 2022. Siberian hamsters nonresponding to short photoperiod use fasting-induced torpor. Journal of Experimental Biology 225(12).

Pusey H, Cooper CE, Withers PC. 2013. Metabolic, hygric and ventilatory physiology of the red-tailed phascogale (Phascogale calura; Marsupialia, Dasyuridae): Adaptations to aridity or arboreality? Mammalian Biology 78(6):397-405.

Randrianambinina B, Rakotondravony D, Radespiel U, Zimmermann E. 2003. Seasonal changes in general activity, body mass and reproduction of two small nocturnal primates: a comparison of the golden brown mouse lemur (*Microcebus ravelobensis*) in Northwestern Madagascar and the brown mouse lemur (*Microcebus rufus*) in Eastern Madagascar. Primates 44(4):321-331.

Rasweiler J. 1973. Care and management of the long-tongued bat, *Glossophaga soricina* (Chiroptera: Phyllostomatidae), in the laboratory, with observations on estivation induced by food deprivation. Journal of Mammalogy 54:391–404.

Reardon M. 1999. Quolls on the run. Australian Geographic 54:89–105.

Reher S, Dausmann KH. 2021. Tropical bats counter heat by combining torpor with adaptive hyperthermia. Proc Biol Sci 288(1942):20202059.

Reher S, Ehlers J, Rabarison H, Dausmann KH. 2018. Short and hyperthermic torpor responses in the Malagasy bat Macronycteris commersoni reveal a broader hypometabolic scope in heterotherms. Journal of Comparative Physiology B 188(6):1015-1027.

Reinhard K. 2019. Ecophysiology of a wild nocturnal primate, the Javan slow loris (*Nycticebus javanicus*). Oxford Brookes University.

Renninger M, Sprau L, Geiser F. 2020. White mouse pups can use torpor for energy conservation. Journal of Comparative Physiology B 190(2):253-259.

Riedesel ML, Williams BA. 1976. Continuous 24-hour oxygen consumption studies of *Myotis velifer*. Comp Biochem Physiol A Comp Physiol 54(1):95-9.

Riek A, Körtner G, Geiser F. 2010. Thermobiology, energetics and activity patterns of the Eastern tube-nosed bat (*Nyctimene robinsoni*) in the Australian tropics: effect of temperature and lunar cycle. J Exp Biol 213(Pt 15):2557-64.

Ruf T, Stieglitz A, Steinlechner S, Blank J, Heldmaier G. 1993. Cold exposure and food restriction facilitate physiological responses to short photoperiod in Djungarian hamsters (*Phodopus sungorus*). Journal of Experimental Zoology Part A: Ecological Genetics and Physiology 267:104-112.

Ruf T, Streicher U, Stalder GL, Nadler T, Walzer C. 2015. Hibernation in the pygmy slow loris (*Nycticebus pygmaeus*): multiday torpor in primates is not restricted to Madagascar. Scientific Reports 5:17392.

Rutovskaya M, Diatroptov M. 2022. Seasonal and diurnal variability of the body temperature in the northern white-breasted hedgehog (*Erinaceus roumanicus*) in normothermia. Russian Journal of Theriology 21:103-114.

Rutovskaya M, Diatroptov M, Kuznetsova E, Anufrief A, Feoktistova N, Surov A. 2019. The phenomenon of negative body temperature on hibernating hedgehogs of the genus Erinaceus Journal of Evololutionary Biochemistry and Physiolology 55:515–516.

Salinas R, Valeria B., Herrera M, L. Gerardo, Flores-Martínez JJ, Johnston DS. 2014. Winter and summer torpor in a free-ranging subtropical desert bat: The fishing myotis (*Myotis vivesi*). Acta Chiropterologica 16(2):327-336, 10.

Scantlebury M, Lovegrove B, Jackson C, Bennett N, Lutermann H. 2008. Hibernation and non-shivering thermogenesis in the Hottentot golden mole (*Amblysomus hottentottus longiceps*). Journal of Comparative Physiology B: Biochemical, Systemic, and Environmental Physiology 178(7):887-897.

Schmid J, Ruf J, Heldmaier G. 2000. Metabolism and temperature regulation during daily torpor in the smallest primate, the pygmy mouse lemur (*Microcebus myoxinus*) in Madagascar. Journal of Comparative Physiology B 170:59-68.

Schmid J, Speakman JR. 2000. Daily energy expenditure of the grey mouse lemur (*Microcebus murinus*): a small primate that uses torpor. Journal of Comparative Physiology B 170(8):633-641.

Schubert KA, Boerema AS, Vaanholt LM, de Boer SF, Strijkstra AM, Daan S. 2010. Daily torpor in mice: high foraging costs trigger energy-saving hypothermia. Biol Lett 6(1):132-5.

Seymour RS, Withers PC, Weathers WW. 1998. Energetics of burrowing, running, and free-living in the Namib Desert golden mole (Eremitalpa namibensis). Journal of Zoology 244(1):107-117.

Siutz C, Ammann V, Millesi E. 2018. Shallow Torpor Expression in Free-Ranging Common Hamsters With and Without Food Supplements. Frontiers in Ecology and Evolution 6.

Smith A. 1980. The diet and ecology of leadbeaters possum and the sugar glider. Monash University, Melbourne.

Smith A, Xie Y. 2008. A guide to the mammals of China Princeton University Press

Sørås R, Fjelldal MA, Bech C, van der Kooij J, Skåra KH, Eldegard K, Stawski C. 2022. State dependence of arousal from torpor in brown long-eared bats (*Plecotus auritus*). Journal of Comparative Physiology B 192(6):815-827.

Soriano PJ, Ruiz A, Arends A. 2002. Physiological responses to ambient temperature manipulation by three species of bats from Andean cloud forests. Journal of Mammalogy 83(2):445-457.

Stawski C, Currie SE. 2016. Effect of roost choice on winter torpor patterns of a free-ranging insectivorous bat. Australian Journal of Zoology 64(2):132-137.

Stawski C, Geiser F. 2010. Fat and fed: frequent use of summer torpor in a subtropical bat. Naturwissenschaften 97(1):29-35.

Stawski C, Geiser F. 2012. Will temperature effects or phenotypic plasticity determine the thermal response of a heterothermic tropical bat to climate change? PLOS ONE 7(7):e40278.

Stawski C, Körtner G, Nowack J, Geiser F. 2015. The importance of mammalian torpor for survival in a post-fire landscape. Biology Letters 11(6).

Stawski C, Körtner G, Nowack J, Geiser F. 2016. Phenotypic plasticity of post-fire activity and thermal biology of a free-ranging small mammal. Physiology & Behavior 159:104-111.

Stawski C, Rojas AD. 2016. Thermal physiology of a reproductive female marsupial, *Antechinus flavipes*. Mammal Research:1-5.

Stawski C, Turbill C, Geiser F. 2009. Hibernation by a free-ranging subtropical bat (Nyctophilus bifax). Journal of Comparative Physiology, B: Biochemical, Systematic, and Environmental Physiology 179:433-441.

Stephenson PJ, Racey PA. 1993a. Reproductive energetics of the Tenrecidae (Mammalia: Insectivora). I. The large-eared tenrec, *Geogale aurita*. Physiological Zoology 66(5):643-663.

Stephenson PJ, Racey PA. 1993b. Reproductive energetics of the Tenrecidae (Mammalia: Insectivora). II. The shrew-tenrecs, *Microgale spp*. Physiological Zoology 66(5):664-685.

Stephenson PJ, Racey PA. 1994. Seasonal variation in resting metabolic rate and body temperature of streaked tenrecs, *Hemicentetes nigriceps* and *H. semispinosus* (Insectivora: Tenrecidae). Journal of Zoology 232(2):285-294.

Streicher S. 2010. The effect of environmental variables on patterns of body temperature in the Damaraland mole-rat, *Fukomys damarensis* (Ogilby 1838). University of Pretoria.

Streicher S, Boyles JG, Oosthuizen MK, Bennett NC. 2011. Body temperature patterns and rhythmicity in free-ranging subterranean Damaraland mole-rats, Fukomys damarensis. PLoS One 6(10):e26346.

Strumwasser F. 1960. Some physiological principles governing hibernation in *Citellus beechey*i. Bulletin of the Museum of Comparative Zoology 124:285-320.

Superina M, Boily P. 2007. Hibernation and daily torpor in an armadillo, the pichi (Zaedyus pichiy). Comparative Biochemistry and Physiology Part A: Molecular & Integrative Physiology 148(4):893-898.

Symes SA, Klafki R, Packham R, Larsen KW. 2019. Winter activity patterns of the North American badger (Taxidea taxus) at its northwestern periphery. Journal of Mammalogy 101(1):199-210.

Tanaka H. 2006. Winter hibernation and body temperature fluctuation in the Japanese badger, *Meles meles anakuma*. Zoological Science 23(11):991-997.

Tannenbaum MG, Pivorun EB. 1984. Differences in daily torpor patterns among three southeastern species of Peromyscus. Journal of Comparative Physiology B 154(3):233-236.

Tannenbaum MG, Pivorun EB. 1988. Seasonal study of daily torpor in southeastern *Peromyscus maniculatus* and *Peromyscus leucopus* from mountains and foothills. Physiological Zoology 61(1):10-16.

Tannenbaum MG, Pivorun EB. 1989. Summer torpor in montane *Peromyscus maniculatus*. The American Midland Naturalist 121(1):194-197.

Terada A, Ibuka N. 2000. Age affects hibernation in Syrian hamsters (*Mesocricetus auratus*). Chronobiol Int 17(5):623-30.

Thompson SD. 1985. Subspecific differences in metabolism, thermoregulation, and torpor in the western harvest mouse *Reithrodontomys megalotis*. Physiological Zoology 58(4):430-444.

Tinkle DW, Patterson IG. 1965. A study of hibernating populations of *Myotis velifer* in northwestern Texas. J Mammal 46(4):612-33.

Tøien Ø, Blake J, Edgar DM, Grahn DA, Heller HC, Barnes BM. 2011. Hibernation in black bears: independence of metabolic suppression from body temperature. Science 331(6019):906-909.

Tomlinson S, Withers PC, Cooper C. 2007. Hypothermia versus torpor in response to cold stress in the native Australian mouse *Pseudomys hermannsburgensis* and the introduced house mouse *Mus musculus*. Comparative Biochemistry and Physiology Part A: Molecular &amp; Integrative Physiology 148(3):645-650.

Tomlinson S, Withers PC, Maloney SK. 2012. Flexibility in thermoregulatory physiology of two dunnarts, Sminthopsis macroura and Sminthopsis ooldea (Marsupialia; Dasyuridae). J Exp Biol 215(Pt 13):2236-46.

Treat MD, Scholer L, Barrett B, Khachatryan A, McKenna AJ, Reyes T, Rezazadeh A, Ronkon CF, Samora D, Santamaria JF et al. . 2018. Extreme physiological plasticity in a hibernating basoendothermic mammal, Tenrec ecaudatus. Journal of Experimental Biology 221(20).

Tucker VA. 1965. Oxygen consumption, thermal conductance, and torpor in the California pocket mouse *Perognathus californicus*. Journal of Cellular and Comparative Physiology 65(3):393-403.

Turbill C. 2006. Thermoregulatory behavior of tree-roosting chocolate wattled bats (*Chalinolobus morio*) during summer and winter. Journal of Mammalogy 87(2):318-323.

Turbill C, Geiser F. 2008. Hibernation by tree-roosting bats. Journal of Comparative Physiology B 178(5):597-605.

Turner JM, Geiser F. 2017. The influence of natural photoperiod on seasonal torpor expression of two opportunistic marsupial hibernators. J Comp Physiol B 187(2):375-383.

Turner JM, Körtner G, Warnecke L, Geiser F. 2012a. Summer and winter torpor use by a free-ranging marsupial. Comparative Biochemistry and Physiology Part A: Molecular & Integrative Physiology 162(3):274-280.

Turner JM, Warnecke L, Körtner G, Geiser F. 2012b. Opportunistic hibernation by a free-ranging marsupial. Journal of Zoology 286(4):277-284.

Tyndale-Biscoe H. 1973. Life of marsupials. London: Edward Arnold.

Vivier L, Van der Merwe M. The incidence of torpor in winter and summer in the Angolan free-tailed bat, *Mops condylurus* (Microchiroptera : Molossidae), in a subtropical environment, Mpumulanga, South Africa.42(1):50-58. Available from: <http://reference.sabinet.co.za/webx/access/electronic_journals/afzoo/afzoo_v42_n1_a7.pdf>

Wallis R. 1976. Torpor in the dasyurid marsupial *Antechinus stuartii*. Comp Biochem Physiol 53A:319-322.

Wang LC-H, Hudson JW. 1970. Some physiological aspects of temperature regulation in the normothermic and torpid hispid pocket mouse, *Perognathus hispidus*. Comparative Biochemistry and Physiology 32(2):275-293.

Wang LCH. 1978. Energetic and field aspects of mammalian torpor: the Richardson's ground squirrel. In: Wang LCH, Hudson JW, editors. Strategies in Cold: Natural Torpidity and Thermogenesis New York: Academic Press. p. 109-145.

Warnecke L, Turner J, Geiser F. 2008. Torpor and basking in a small arid zone marsupial. Naturwissenschaften 95(1):73-78.

Wassmer T, Wollnik F. 1997. Timing of torpor bouts during hibernation in European hamsters (Cricetus cricetus L.). J Comp Physiol B 167(4):270-9.

Watanabe D, Hatase M, Sakamoto S, Koshimoto C, Shinohara A, Morita T. 2016. Torpor capability in two gerbil species, *Meriones unguiculatus* and *Tatera indica*. Japanese Journal of Environmental Entomology and Zoology 27(1):9-16.

Webb PI, Skinner JD. 1996. Summer torpor in African woodland dormice *Graphiurus murinus* (Myoxidae: Graphiurinae). Journal of Comparative Physiology B: Biochemical, Systemic, and Environmental Physiology 166:325-330.

Wein J. 2010. Effects of ambient temperature on tropical hibernation in the lesser hedgehog tenrec, *Echinops telfairi*. [Hamburg, Germany]: University of Hamburg.

Williams CT, Barnes BM, Kenagy GJ, Buck CL. 2014. Phenology of hibernation and reproduction in ground squirrels: integration of environmental cues with endogenous programming. Journal of Zoology 292(2):112-124.

Willis CK, Turbill C, Geiser F. 2005a. Torpor and thermal energetics in a tiny Australian vespertilionid, the little forest bat (*Vespadelus vulturnus*). J Comp Physiol B 175(7):479-86.

Willis CKR, Brigham RM, Geiser F. 2006. Deep, prolonged torpor by pregnant, free-ranging bats. Naturwissenschaften 93(2):80-83.

Willis CKR, Lane JE, Liknes ET, Swanson DL, Brigham RM. 2005b. Thermal energetics of female big brown bats (*Eptesicus fuscus*). Canadian Journal of Zoology 83(6):871-879.

Wilz M, Heldmaier G. 2000. Comparison of hibernation, estivation and daily torpor in the edible dormouse, *Glis glis*. Journal of Comparative Physiology B 170(7):511-521.

Withers P, Louw G, Henschel J. 1980. Energetics and water relations of Namib desert rodents. South African Journal of Zoology 15:131-137.

Withers P, Richardson K, Wooller R. 1989. Metabolic physiology of euthermic and torpid honey possums, *Tarsipes rostratus*. Australian Journal of Zoology 37(6):685-693.

Withers PC, Cooper CE. 2009. Thermal, metabolic, hygric and ventilatory physiology of the sandhill dunnart (Sminthopsis psammophila; Marsupialia, Dasyuridae). Comparative Biochemistry and Physiology Part A: Molecular & Integrative Physiology 153(3):317-323.

Withers PC, Thompson GG, Seymour RS. 2000. Metabolic physiology of the north-western marsupial mole, *Notoryctes caurinus* (Marsupialia : Notoryctidae). Australian Journal of Zoology 48(3):241-258.

Wojciechowski MS, Jefimow M, Tęgowska E. 2007. Environmental conditions, rather than season, determine torpor use and temperature selection in large mouse-eared bats (*Myotis myotis*). Comparative Biochemistry and Physiology Part A: Molecular & Integrative Physiology 147(4):828-840.

Yang M, Xing X, Guan S, Zhao Y, Wang Z, Wang D-H. 2011. Hibernation patterns and changes of body temperature in Daurian ground squirrels (*Spermophilus dauricus)* during hibernation. Acta Theriologica Sinica 31:387-395.

Young PJ. 1990. Hibernating patterns of free-ranging Columbian ground squirrels. Oecologia 83(4):504-511.

Young RA. 2001. The eastern horseshoe bat, *Rhinolophus megaphyllus*, in south-east Queensland, Australia: colony demography and dynamics, activity levels, seasonal weight changes, and capture-recapture analyses. Wildlife Research 28:425-434.

Yousef MK, Dill DB. 1971. Daily cycles of hibernation in the kangaroo rat, *Dipodomys merriami*. Cryobiology 8 5:441-6.

Zervanos SM, Maher CR, Florant GL. 2013. Effect of body mass on hibernation strategies of woodchucks (*Marmota monax*). Integrative and Comparative Biology 54(3):443-451.
